# Supplementary material for: Estimating the natural indirect effect and the mediation proportion via the product method
Source: BMC Med Res Methodol. 2021 Nov 20;21:253. doi: 10.1186/s12874-021-01425-4 (PMC8606099; doi:10.1186/s12874-021-01425-4)
Supplement: Supplementary file 1 — Additional file 1 “Web Material.pdf”: online supplementary material for “Estimating the natural indirect effect and the mediation proportion via the product method”, including Web Appendices A–D, Web Tables 1–8 and Web Figures 1–4 not shown in the manuscript. [file 12874_2021_1425_MOESM1_ESM.pdf]

Web Material for "Estimating the natural indirect effect and the mediation proportion via the product method"

Chao Cheng, Donna Spiegelman, and Fan Li

This documentation presented the web appendices, tables and figures not shown in the manuscript.

1 Web Appendics

Web Appendix A: Causal mediation measures for a binary outcome and continuous mediator

When the outcome is binary, the expectations of the counterfactual outcome in equation (1), $E[Y_{x,M_x}|\boldsymbol{W} = \boldsymbol{w}]$, $E[Y_{x,M_{x^*}}|\boldsymbol{W} = \boldsymbol{w}]$, and $E[Y_{x^*,M_{x^*}}|\boldsymbol{W} = \boldsymbol{w}]$, simplify to $P(Y_{xM_x} = 1|\boldsymbol{W} = \boldsymbol{w})$, $P(Y_{xM_{x^*}} = 1|\boldsymbol{W} = \boldsymbol{w})$ and $P(Y_{x^*M_{x^*}} = 1|\boldsymbol{W} = \boldsymbol{w})$, respectively. Gaynor et al. (2019) derived the expressions of $P(Y_{xM_x} = 1|\boldsymbol{w})$, $P(Y_{xM_{x^*}} = 1|\boldsymbol{w})$ and $P(Y_{x^*M_{x^*}} = 1|\boldsymbol{w})$ while developing the probit approximation method for mediation analysis for common binary outcomes, but did not simply these expressions in their manuscript. Here, we repeated their work and developed a simplified version of mediation measure expressions, with notations in our manuscript. At the end of this section, we compared the exact expressions and approximate expressions under a rare outcome assumption and showed that the exact expressions degenerated to the approximate expressions if the outcome was rare.

If model (1) has a logistic link function and the error term in (4) follows $N(0, \sigma^2)$, $P(Y_{xM_x} = 1|\boldsymbol{W} = \boldsymbol{w})$ is shown as

$$\begin{aligned} P(Y_{xM_x} = 1|\boldsymbol{W} = \boldsymbol{w}) &= \int_m Pr(Y = 1|X = x, M = m, \boldsymbol{W} = \boldsymbol{w}) f(M = m|X = x, \boldsymbol{W} = \boldsymbol{w}) \mathrm{d}m \\ &= \int_m \frac{\exp(\beta_0 + \beta_1 x + \beta_2 m + \boldsymbol{\beta}_3^T \boldsymbol{w})}{1 + \exp(\beta_0 + \beta_1 x + \beta_2 m + \boldsymbol{\beta}_3^T \boldsymbol{w})} \times \frac{1}{\sqrt{2\pi}\sigma^2} \exp\Big(- \frac{(m - \gamma_0 - \gamma_1 x - \boldsymbol{\gamma}_2^T \boldsymbol{w})^2}{2\sigma^2} \Big) \mathrm{d}m. \end{aligned}$$

Therefore,

$$
\begin{aligned}
\operatorname{logit}\Big(P(Y_{xM_x}=1|\boldsymbol{W}=\boldsymbol{w})\Big) &= \log\Big(\frac{P(Y_{xM_x}=1|\boldsymbol{w})}{1-P(Y_{xM_x}=1|\boldsymbol{w})}\Big) \\
&= \log\left\{\frac{\int_m \frac{\exp(\beta_0+\beta_1x+\beta_2m+\boldsymbol{\beta}_3^T\boldsymbol{w})}{1+\exp(\beta_0+\beta_1x+\beta_2m+\boldsymbol{\beta}_3^T\boldsymbol{w})}\times\frac{1}{\sqrt{2\pi\sigma^2}}\exp\Big(-\frac{(m-\gamma_0-\gamma_1x-\boldsymbol{\gamma}_2^T\boldsymbol{w})^2}{2\sigma^2}\Big)\mathrm{d}m}{\int_m \frac{1}{1+\exp(\beta_0+\beta_1x+\beta_2m+\boldsymbol{\beta}_3^T\boldsymbol{w})}\times\frac{1}{\sqrt{2\pi\sigma^2}}\exp\Big(-\frac{(m-\gamma_0-\gamma_1x-\boldsymbol{\gamma}_2^T\boldsymbol{w})^2}{2\sigma^2}\Big)\mathrm{d}m}\right) \\
&= \log\left\{\frac{e^{\beta_0+\beta_1x+\boldsymbol{\beta}_3^T\boldsymbol{w}-\frac{(\gamma_0+\gamma_1x+\boldsymbol{\gamma}_2^T\boldsymbol{w})^2}{2\sigma^2}}\int_m\frac{\exp(\beta_2m-\frac{m^2-2(\gamma_0+\gamma_1x+\boldsymbol{\gamma}_2^T\boldsymbol{w})m}{2\sigma^2})}{1+\exp(\beta_0+\beta_1x+\beta_2m+\boldsymbol{\beta}_3^T\boldsymbol{w})}\mathrm{d}m}{e^{-\frac{(\gamma_0+\gamma_1x+\boldsymbol{\gamma}_2^T\boldsymbol{w})^2}{2\sigma^2}}\int_m\frac{\exp(-\frac{m^2-2(\gamma_0+\gamma_1x+\boldsymbol{\gamma}_2^T\boldsymbol{w})m}{2\sigma^2})}{1+\exp(\beta_0+\beta_1x+\beta_2m+\boldsymbol{\beta}_3^T\boldsymbol{w})}\mathrm{d}m}\right\} \\
&= \beta_0+\beta_1x+\boldsymbol{\beta}_3^T\boldsymbol{w}+\log\left\{\frac{\int_m\frac{\exp(-\frac{m^2-2(\sigma^2\beta_2+\gamma_0+\gamma_1x+\boldsymbol{\gamma}_2^T\boldsymbol{w})m}{2\sigma^2})}{1+\exp(\beta_0+\beta_1x+\beta_2m+\boldsymbol{\beta}_3^T\boldsymbol{w})}\mathrm{d}m}{\int_m\frac{\exp(-\frac{m^2-2(\gamma_0+\gamma_1x+\boldsymbol{\gamma}_2^T\boldsymbol{w})m}{2\sigma^2})}{1+\exp(\beta_0+\beta_1x+\beta_2m+\boldsymbol{\beta}_3^T\boldsymbol{w})}\mathrm{d}m}\right\}
\end{aligned}
$$

If we define

$$
\tau(x',x,m,\boldsymbol{w})=\frac{\exp\{(\gamma_0+\gamma_1x'+\boldsymbol{\gamma}_2^T\boldsymbol{w})m/\sigma^2-m^2/(2\sigma^2)\}}{1+\exp(\beta_0+\beta_1x+\beta_2m+\boldsymbol{\beta}_3^T\boldsymbol{w})},
$$

then the above quantity simplifies to

$$
P(Y_{xM_x}=1|\boldsymbol{W}=\boldsymbol{w})=\beta_0+\beta_1x+\boldsymbol{\beta}_3^T\boldsymbol{w}+\log\left\{\frac{\int_m\exp(\beta_2m)\tau(x,x,m,\boldsymbol{w})\mathrm{d}m}{\int_m\tau(x,x,m,\boldsymbol{w})\mathrm{d}m}\right\}
$$

Similarly, we can show

$$
\operatorname{logit}\Big(P(Y_{xM_{x^*}}=1|\boldsymbol{W}=\boldsymbol{w})\Big)=\beta_0+\beta_1x+\boldsymbol{\beta}_3^T\boldsymbol{w}+\log\left\{\frac{\int_m\exp(\beta_2m)\tau(x^*,x,m,\boldsymbol{w})\mathrm{d}m}{\int_m\tau(x^*,x,m,\boldsymbol{w})\mathrm{d}m}\right\},
$$

$$
\operatorname{logit}\Big(P(Y_{x^*M_{x^*}}=1|\boldsymbol{W}=\boldsymbol{w})\Big)=\beta_0+\beta_1x^*+\boldsymbol{\beta}_3^T\boldsymbol{w}+\log\left\{\frac{\int_m\exp(\beta_2m)\tau(x^*,x^*,m,\boldsymbol{w})\mathrm{d}m}{\int_m\tau(x^*,x^*,m,\boldsymbol{w})\mathrm{d}m}\right\}.
$$

Finally, with a logistic link, the NIE, NDE and TE conditional on $\boldsymbol{W}=\boldsymbol{w}$ for a change in X from x^* to x is

$$
\begin{aligned}
\mathrm{NIE} &= \operatorname{logit}\Big(P(Y_{xM_x}=1|\boldsymbol{W}=\boldsymbol{w})\Big)-\operatorname{logit}\Big(P(Y_{xM_{x^*}}=1|\boldsymbol{W}=\boldsymbol{w})\Big) \\
&= \log\left\{\frac{\int_m\exp(\beta_2m)\tau(x,x,m,\boldsymbol{w})\mathrm{d}m}{\int_m\tau(x,x,m,\boldsymbol{w})\mathrm{d}m}\right\}-\log\left\{\frac{\int_m\exp(\beta_2m)\tau(x^*,x,m,\boldsymbol{w})\mathrm{d}m}{\int_m\tau(x^*,x,m,\boldsymbol{w})\mathrm{d}m}\right\}, \\
\mathrm{NDE} &= \operatorname{logit}\Big(P(Y_{xM_{x^*}}=1|\boldsymbol{W}=\boldsymbol{w})\Big)-\operatorname{logit}\Big(P(Y_{x^*M_{x^*}}=1|\boldsymbol{W}=\boldsymbol{w})\Big) \\
&= \beta_1(x-x^*)+\log\left\{\frac{\int_m\exp(\beta_2m)\tau(x^*,x,m,\boldsymbol{w})\mathrm{d}m}{\int_m\tau(x^*,x,m,\boldsymbol{w})\mathrm{d}m}\right\}-\log\left\{\frac{\int_m\exp(\beta_2m)\tau(x^*,x^*,m,\boldsymbol{w})\mathrm{d}m}{\int_m\tau(x^*,x^*,m,\boldsymbol{w})\mathrm{d}m}\right\}, \\
\mathrm{TE} &= \operatorname{logit}\Big(P(Y_{xM_x}=1|\boldsymbol{W}=\boldsymbol{w})\Big)-\operatorname{logit}\Big(P(Y_{x^*M_{x^*}}=1|\boldsymbol{W}=\boldsymbol{w})\Big) \\
&= \beta_1(x-x^*)+\log\left\{\frac{\int_m\exp(\beta_2m)\tau(x,x,m,\boldsymbol{w})\mathrm{d}m}{\int_m\tau(x,x,m,\boldsymbol{w})\mathrm{d}m}\right\}-\log\left\{\frac{\int_m\exp(\beta_2m)\tau(x^*,x^*,m,\boldsymbol{w})\mathrm{d}m}{\int_m\tau(x^*,x^*,m,\boldsymbol{w})\mathrm{d}m}\right\}.
\end{aligned}
$$

As always, $\mathrm{MP}=\frac{\mathrm{NIE}}{\mathrm{TE}}$. Substituting the point estimates of $\boldsymbol{\gamma}$, $\boldsymbol{\beta}$ and σ^2 into the above expressions leads to $\widehat{\mathrm{NIE}}$, $\widehat{\mathrm{NDE}}$, $\widehat{\mathrm{TE}}$, and $\widehat{\mathrm{MP}}$. Because the integrals in the above expressions do not have closed-form solutions, we

can use some integrate methods to numerically calculate the integrals, such as the Gauss-Hermite Quadrature (abbreviated by GHQ, Liu and Pierce, 1994) and the QUADPACK approach (Piessens et al., 2012). When using 30 or more knots in the GHQ approach and setting the relative accuracy in the QUADPACK to 10^{-4} or less, we observed that both integrate approaches performed very close for calculating NIE and MP estimates, where the differences of their calculations were generally less than 10^{-7} under the parameter settings of $\boldsymbol{\gamma}$, $\boldsymbol{\beta}$ and σ^2 in our simulation studies. Therefore, we only used the GHQ method in the simulation studies and our R package.

Instead of calculating the integrals numerically, Gaynor et al. (2019) used a probit function to approximate the logit function in the integrals and obtained closed-form expressions for the mediation measures (see Table 2 in the manuscript for the expressions). However, this probit approximation method could be crude as the outcome prevalence further deviates from 50%, as discussed in Gaynor et al. (2019). We conducted a brief numerical study to compare the percent bias of the probit approximation method for computing the NIE and MP, where the numerical integral calculation based on the GHQ were treated as the gold standard. We set TE and MP, defined for X in change from 0 to 1, as log(1.5) and 0.5, respectively, while changing the baseline outcome prevalence from 1% to 95%, where other settings and parameter values are same to the simulation study section. Web Figure 1 visualized the percent bias of the probit approximation approach. Although the precent bias for MP were minimal, the probit approximation method exhibited high bias for calculating NIE if the baseline prevalence is less than 10% or greater than 80%, where the percent bias exceeds 40% when outcome prevalences below 5%.

Generally, the above expressions for NIE, NDE and TE can not be further simplified, as the integrals in the expressions do not have closed forms. However, if the outcome is sufficient rare, the denominator in $\tau(x', x, m, \boldsymbol{w})$, i.e., $1 + \exp(\beta_0 + \beta_1 x + \beta_2 m + \boldsymbol{\beta}_3^T \boldsymbol{w})$, approximates to 1. If we apply this approximation to the NIE expression, we have

$$
\begin{aligned}
\text{NIE} \approx & \log\left(\frac{\int_m \exp(-\frac{m^2-2(\sigma^2\beta_2+\gamma_0+\gamma_1 x+\boldsymbol{\gamma}_2^T\boldsymbol{w})m}{2\sigma^2})\mathrm{d}m}{\int_m \exp(-\frac{m^2-2(\gamma_0+\gamma_1 x+\boldsymbol{\gamma}_2^T\boldsymbol{w})m}{2\sigma^2})\mathrm{d}m}\right) - \log\left(\frac{\int_m \exp(-\frac{m^2-2(\sigma^2\beta_2+\gamma_0+\gamma_1 x^*+\boldsymbol{\gamma}_2^T\boldsymbol{w})m}{2\sigma^2})\mathrm{d}m}{\int_m \exp(-\frac{m^2-2(\gamma_0+\gamma_1 x^*+\boldsymbol{\gamma}_2^T\boldsymbol{w})m}{2\sigma^2})\mathrm{d}m}\right) \\
= & \log\left(\frac{\int_m \exp(-\frac{\left(m-(\sigma^2\beta_2+\gamma_0+\gamma_1 x+\boldsymbol{\gamma}_2^T\boldsymbol{w})\right)^2}{2\sigma^2})\mathrm{d}m \times \exp\left(\frac{(\beta_2\sigma^2+\gamma_0+\gamma_1 x+\boldsymbol{\gamma}_2^T\boldsymbol{w})^2}{2\sigma^2}\right)}{\int_m \exp(-\frac{\left(m-(\gamma_0+\gamma_1 x+\boldsymbol{\gamma}_2^T\boldsymbol{w})\right)^2}{2\sigma^2})\mathrm{d}m \times \exp\left(\frac{(\gamma_0+\gamma_1 x+\boldsymbol{\gamma}_2^T\boldsymbol{w})^2}{2\sigma^2}\right)}\right) \\
& - \log\left(\frac{\int_m \exp(-\frac{\left(m-(\sigma^2\beta_2+\gamma_0+\gamma_1 x^*+\boldsymbol{\gamma}_2^T\boldsymbol{w})\right)^2}{2\sigma^2})\mathrm{d}m \times \exp\left(\frac{(\beta_2\sigma^2+\gamma_0+\gamma_1 x^*+\boldsymbol{\gamma}_2^T\boldsymbol{w})^2}{2\sigma^2}\right)}{\int_m \exp(-\frac{\left(m-(\gamma_0+\gamma_1 x^*+\boldsymbol{\gamma}_2^T\boldsymbol{w})\right)^2}{2\sigma^2})\mathrm{d}m \times \exp\left(\frac{(\gamma_0+\gamma_1 x^*+\boldsymbol{\gamma}_2^T\boldsymbol{w})^2}{2\sigma^2}\right)}\right) \\
= & \log\left(\frac{\sqrt{2\pi\sigma^2} \times \exp\left(\frac{(\beta_2\sigma^2+\gamma_0+\gamma_1 x+\boldsymbol{\gamma}_2^T\boldsymbol{w})^2}{2\sigma^2}\right)}{\sqrt{2\pi\sigma^2} \times \exp\left(\frac{(\gamma_0+\gamma_1 x+\boldsymbol{\gamma}_2^T\boldsymbol{w})^2}{2\sigma^2}\right)}\right) - \log\left(\frac{\sqrt{2\pi\sigma^2} \times \exp\left(\frac{(\beta_2\sigma^2+\gamma_0+\gamma_1 x^*+\boldsymbol{\gamma}_2^T\boldsymbol{w})^2}{2\sigma^2}\right)}{\sqrt{2\pi\sigma^2} \times \exp\left(\frac{(\gamma_0+\gamma_1 x^*+\boldsymbol{\gamma}_2^T\boldsymbol{w})^2}{2\sigma^2}\right)}\right) \\
= & \beta_2\gamma_1(x - x^*).
\end{aligned}
$$

Using a similar strategy, we can show NDE $\approx \beta_1(x - x^*)$ and TE $\approx (\beta_2\gamma_1 + \beta_1)(x - x^*)$. As a result MP $\approx \frac{\beta_2\gamma_1}{\beta_2\gamma_1+\beta_1}$.

Web Appendix B: Causal mediation measures under binary outcome and binary mediator

As given in Gaynor et al. (2019), $P(Y_{xM_x}=1|\boldsymbol{W}=\boldsymbol{w})$, $P(Y_{xM_{x^*}}=1|\boldsymbol{W}=\boldsymbol{w})$, and $P(Y_{x^*M_{x^*}}=1|\boldsymbol{W}=\boldsymbol{w})$ can be shown as

$$
\begin{aligned}
P(Y_{xM_x}=1|\boldsymbol{W}=\boldsymbol{w}) &= \sum_{m=0}^{1} P(Y=1|x,m,\boldsymbol{w})P(M=m|x,\boldsymbol{w}) \\
&= \frac{\exp(\beta_0+\beta_1+\boldsymbol{\beta}_3^T\boldsymbol{w})}{1+\exp(\beta_0+\beta_1+\boldsymbol{\beta}_3^T\boldsymbol{w})}\times\frac{1}{1+\exp(\gamma_0+\gamma_1+\boldsymbol{\gamma}_2^T\boldsymbol{w})}+\frac{\exp(\beta_0+\beta_1+\beta_2+\boldsymbol{\beta}_3^T\boldsymbol{w})}{1+\exp(\beta_0+\beta_1+\beta_2+\boldsymbol{\beta}_3^T\boldsymbol{w})}\times\frac{\exp(\gamma_0+\gamma_1+\boldsymbol{\gamma}_2^T\boldsymbol{w})}{1+\exp(\gamma_0+\gamma_1+\boldsymbol{\gamma}_2^T\boldsymbol{w})}, \\
P(Y_{xM_{x^*}}=1|\boldsymbol{W}=\boldsymbol{w}) &= \sum_{m=0}^{1} P(Y=1|x,m,\boldsymbol{w})P(M=m|x^*,\boldsymbol{w}) \\
&= \frac{\exp(\beta_0+\beta_1+\boldsymbol{\beta}_3^T\boldsymbol{w})}{1+\exp(\beta_0+\beta_1+\boldsymbol{\beta}_3^T\boldsymbol{w})}\times\frac{1}{1+\exp(\gamma_0+\boldsymbol{\gamma}_2^T\boldsymbol{w})}+\frac{\exp(\beta_0+\beta_1+\beta_2+\boldsymbol{\beta}_3^T\boldsymbol{w})}{1+\exp(\beta_0+\beta_1+\beta_2+\boldsymbol{\beta}_3^T\boldsymbol{w})}\times\frac{\exp(\gamma_0+\boldsymbol{\gamma}_2^T\boldsymbol{w})}{1+\exp(\gamma_0+\boldsymbol{\gamma}_2^T\boldsymbol{w})}, \\
P(Y_{x^*M_{x^*}}=1|\boldsymbol{W}=\boldsymbol{w}) &= \sum_{m=0}^{1} P(Y=1|x^*,m,\boldsymbol{w})P(M=m|x^*,\boldsymbol{w}) \\
&= \frac{\exp(\beta_0+\boldsymbol{\beta}_3^T w)}{1+\exp(\beta_0+\boldsymbol{\beta}_3^T w)}\times\frac{1}{1+\exp(\gamma_0+\boldsymbol{\gamma}_2^T\boldsymbol{w})}+\frac{\exp(\beta_0+\beta_2+\boldsymbol{\beta}_3^T\boldsymbol{w})}{1+\exp(\beta_0+\beta_2+\boldsymbol{\beta}_3^T\boldsymbol{w})}\times\frac{\exp(\gamma_0+\boldsymbol{\gamma}_2^T\boldsymbol{w})}{1+\exp(\gamma_0+\boldsymbol{\gamma}_2^T\boldsymbol{w})}.
\end{aligned}
$$

Next, substituting above probabilities into the expressions of NIE, NDE and TE leads to

$$
\begin{aligned}
\text{NIE} &= \log\left\{\frac{P(Y_{xM_x}=1|\boldsymbol{W}=\boldsymbol{w})/\Big(1-P(Y_{xM_x}=1|\boldsymbol{W}=\boldsymbol{w})\Big)}{P(Y_{xM_{x^*}}=1|\boldsymbol{W}=\boldsymbol{w})/\Big(1-P(Y_{xM_{x^*}}=1|\boldsymbol{W}=\boldsymbol{w})\Big)}\right\} \\
&= \log\left\{\frac{1+e^{\beta_0+\beta_1x+\beta_2+\boldsymbol{\beta}_3^T\boldsymbol{w}}+e^{\gamma_0+\gamma_1x^*+\boldsymbol{\gamma}_2^T\boldsymbol{w}}\big(1+e^{\beta_0+\beta_1x+\boldsymbol{\beta}_3^T\boldsymbol{w}}\big)}{1+e^{\beta_0+\beta_1x+\beta_2+\boldsymbol{\beta}_3^T\boldsymbol{w}}+e^{\gamma_0+\gamma_1x+\boldsymbol{\gamma}_2^T\boldsymbol{w}}\big(1+e^{\beta_0+\beta_1x+\boldsymbol{\beta}_3^T\boldsymbol{w}}\big)}\right\} \\
&\quad+\log\left\{\frac{1+e^{\beta_0+\beta_1x+\beta_2+\boldsymbol{\beta}_3^T\boldsymbol{w}}+e^{\beta_2+\gamma_0+\gamma_1x+\boldsymbol{\gamma}_2^T\boldsymbol{w}}\big(1+e^{\beta_0+\beta_1x+\boldsymbol{\beta}_3^T\boldsymbol{w}}\big)}{1+e^{\beta_0+\beta_1x+\beta_2+\boldsymbol{\beta}_3^T\boldsymbol{w}}+e^{\beta_2+\gamma_0+\gamma_1x^*+\boldsymbol{\gamma}_2\boldsymbol{w}}\big(1+e^{\beta_0+\beta_1x+\boldsymbol{\beta}_3^T\boldsymbol{w}}\big)}\right\},
\end{aligned}
$$

$$
\begin{aligned}
\text{NDE} &= \log\left\{\frac{P(Y_{xM_x^*}=1|\boldsymbol{W}=\boldsymbol{w})/\Big(1-P(Y_{xM_{x^*}}=1|\boldsymbol{W}=\boldsymbol{w})\Big)}{P(Y_{x^*M_{x^*}}=1|\boldsymbol{W}=\boldsymbol{w})/\Big(1-P(Y_{x^*M_{x^*}}=1|\boldsymbol{W}=\boldsymbol{w})\Big)}\right\} \\
&= \beta_1(x-x^*)+\log\left\{\frac{1+e^{\beta_0+\beta_1x^*+\beta_2+\boldsymbol{\beta}_3^T\boldsymbol{w}}+e^{\gamma_0+\gamma_1x^*+\boldsymbol{\gamma}_2^T\boldsymbol{w}}\big(1+e^{\beta_0+\beta_1x^*+\boldsymbol{\beta}_3^T\boldsymbol{w}}\big)}{1+e^{\beta_0+\beta_1x+\beta_2+\boldsymbol{\beta}_3^T\boldsymbol{w}}+e^{\gamma_0+\gamma_1x^*+\boldsymbol{\gamma}_2^T\boldsymbol{w}}\big(1+e^{\beta_0+\beta_1x+\boldsymbol{\beta}_3^T\boldsymbol{w}}\big)}\right\} \\
&\quad+\log\left\{\frac{1+e^{\beta_0+\beta_1x+\beta_2+\boldsymbol{\beta}_3^T\boldsymbol{w}}+e^{\beta_2+\gamma_0+\gamma_1x^*+\boldsymbol{\gamma}_2^T\boldsymbol{w}}\big(1+e^{\beta_0+\beta_1x+\boldsymbol{\beta}_3^T\boldsymbol{w}}\big)}{1+e^{\beta_0+\beta_1x^*+\beta_2+\boldsymbol{\beta}_3^T\boldsymbol{w}}+e^{\beta_2+\gamma_0+\gamma_1x^*+\boldsymbol{\gamma}_2^T\boldsymbol{w}}\big(1+e^{\beta_0+\beta_1x^*+\boldsymbol{\beta}_3^T\boldsymbol{w}}\big)}\right\},
\end{aligned}
$$

and

$$\begin{aligned}
\text{TE} =& \log\left\{\frac{P(Y_{xM_x}=1|\boldsymbol{W}=\boldsymbol{w})/\Big(1-P(Y_{xM_x}=1|\boldsymbol{W}=\boldsymbol{w})\Big)}{P(Y_{x^*M_{x^*}}=1|\boldsymbol{W}=\boldsymbol{w})/\Big(1-P(Y_{x^*M_{x^*}}=1|\boldsymbol{W}=\boldsymbol{w})\Big)}\right\}\\
=&\beta_1(x-x^*)+\log\left\{\frac{1+e^{\beta_0+\beta_1x^*+\beta_2+\boldsymbol{\beta}_3^T\boldsymbol{w}}+e^{\gamma_0+\gamma_1x^*+\boldsymbol{\gamma}_2^T\boldsymbol{w}}(1+e^{\beta_0+\beta_1x^*+\boldsymbol{\beta}_3^T\boldsymbol{w}})}{1+e^{\beta_0+\beta_1x+\beta_2+\boldsymbol{\beta}_3^T\boldsymbol{w}}+e^{\gamma_0+\gamma_1x+\boldsymbol{\gamma}_2^T\boldsymbol{w}}(1+e^{\beta_0+\beta_1x+\boldsymbol{\beta}_3^T\boldsymbol{w}})}\right\}\\
&+\log\left\{\frac{1+e^{\beta_0+\beta_1x+\beta_2+\boldsymbol{\beta}_3^T\boldsymbol{w}}+e^{\beta_2+\gamma_0+\gamma_1x+\boldsymbol{\gamma}_2^T\boldsymbol{w}}(1+e^{\beta_0+\beta_1x+\boldsymbol{\beta}_3^T\boldsymbol{w}})}{1+e^{\beta_0+\beta_1x^*+\beta_2+\boldsymbol{\beta}_3^T\boldsymbol{w}}+e^{\beta_2+\gamma_0+\gamma_1x^*+\boldsymbol{\gamma}_2^T\boldsymbol{w}}(1+e^{\beta_0+\beta_1x^*+\boldsymbol{\beta}_3^T\boldsymbol{w}})}\right\}.
\end{aligned}$$

If we define $\kappa(x,\boldsymbol{w})=\exp(\gamma_0+\gamma_1x+\boldsymbol{\gamma}_2^T\boldsymbol{w})$ and $\eta(x,\boldsymbol{w})=\exp(\beta_0+\beta_1x+\beta_3^T\boldsymbol{w})$, NIE, NDE, and TE simplifies to

$$\begin{aligned}
\text{NIE}=&\log\left\{\frac{1+e^{\beta_2}\eta(x,\boldsymbol{w})+\kappa(x^*,\boldsymbol{w})(1+\eta(x,\boldsymbol{w}))}{1+e^{\beta_2}\eta(x,\boldsymbol{w})+\kappa(x,\boldsymbol{w})(1+\eta(x,\boldsymbol{w}))}\right\}\\
&+\log\left\{\frac{1+e^{\beta_2}\eta(x,\boldsymbol{w})+e^{\beta_2}\kappa(x,\boldsymbol{w})(1+\eta(x,\boldsymbol{w}))}{1+e^{\beta_2}\eta(x,\boldsymbol{w})+e^{\beta_2}\kappa(x^*,\boldsymbol{w})(1+\eta(x,\boldsymbol{w}))}\right\}\\
\text{NDE}=&\beta_1(x-x^*)+\log\left\{\frac{1+e^{\beta_2}\eta(x^*,\boldsymbol{w})+\kappa(x^*,\boldsymbol{w})(1+\eta(x^*,\boldsymbol{w}))}{1+e^{\beta_2}\eta(x,\boldsymbol{w})+\kappa(x^*,\boldsymbol{w})(1+\eta(x,\boldsymbol{w}))}\right\}\\
&+\log\left\{\frac{1+e^{\beta_2}\eta(x,\boldsymbol{w})+e^{\beta_2}\kappa(x^*,\boldsymbol{w})(1+\eta(x,\boldsymbol{w}))}{1+e^{\beta_2}\eta(x^*,\boldsymbol{w})+e^{\beta_2}\kappa(x^*,\boldsymbol{w})(1+\eta(x^*,\boldsymbol{w}))}\right\}\\
\text{TE}=&\beta_1(x-x^*)+\log\left\{\frac{1+e^{\beta_2}\eta(x^*,\boldsymbol{w})+\kappa(x^*,\boldsymbol{w})(1+\eta(x^*,\boldsymbol{w}))}{1+e^{\beta_2}\eta(x,\boldsymbol{w})+\kappa(x,\boldsymbol{w})(1+\eta(x,\boldsymbol{w}))}\right\}\\
&+\log\left\{\frac{1+e^{\beta_2}\eta(x,\boldsymbol{w})+e^{\beta_2}\kappa(x,\boldsymbol{w})(1+\eta(x,\boldsymbol{w}))}{1+e^{\beta_2}\eta(x^*,\boldsymbol{w})+e^{\beta_2}\kappa(x^*,\boldsymbol{w})(1+\eta(x^*,\boldsymbol{w}))}\right\}
\end{aligned}$$

Note that the expressions of NIE, NDE and TE are simple combinations of parameters in the outcome and mediator regression models. The MP on a log odds ratio scale is given by $\frac{\text{NIE}}{\text{TE}}$.

When the outcome is rare, β_0 is a very small value approaching $-\infty$, making $\eta(x,\boldsymbol{w})=\exp(\beta_0+\beta_1x+\beta_3^T\boldsymbol{w})\approx 0$ and $\eta(x^*,\boldsymbol{w})=\exp(\beta_0+\beta_1x^*+\beta_3^T\boldsymbol{w})\approx 0$. Applying those approximations simplifies to the approximated versions of NIE, NDE and TE under a rare outcome assumption

$$\begin{aligned}
\text{NIE}^{(a)}=&\log\left\{\frac{(1+\kappa(x^*,\boldsymbol{w}))(1+e^{\beta_2}\kappa(x,\boldsymbol{w}))}{(1+\kappa(x,\boldsymbol{w}))(1+e^{\beta_2}\kappa(x^*,\boldsymbol{w}))}\right\},\\
\text{NDE}^{(a)}=&\beta_1(x-x^*),\\
\text{TE}^{(a)}=&\beta_1(x-x^*)+\log\left\{\frac{(1+\kappa(x^*,\boldsymbol{w}))(1+e^{\beta_2}\kappa(x,\boldsymbol{w}))}{(1+\kappa(x,\boldsymbol{w}))(1+e^{\beta_2}\kappa(x^*,\boldsymbol{w}))}\right\}.
\end{aligned}$$

It follows that $\text{MP}^{(a)}=\frac{\log\left\{\frac{(1+\kappa(x^*,\boldsymbol{w}))(1+e^{\beta_2}\kappa(x,\boldsymbol{w}))}{(1+\kappa(x,\boldsymbol{w}))(1+e^{\beta_2}\kappa(x^*,\boldsymbol{w}))}\right\}}{\beta_1(x-x^*)+\log\left\{\frac{(1+\kappa(x^*,\boldsymbol{w}))(1+e^{\beta_2}\kappa(x,\boldsymbol{w}))}{(1+\kappa(x,\boldsymbol{w}))(1+e^{\beta_2}\kappa(x^*,\boldsymbol{w}))}\right\}}$.

Web Appendix C: Proof for Result 1

For $\hat{\boldsymbol{\beta}}$ obtained by solving $U(\boldsymbol{\beta}) = 0$, we can expand the estimating equation around the true parameter value

$$\begin{aligned}
\sqrt{n}(\hat{\boldsymbol{\beta}} - \boldsymbol{\beta}) &= -\left[\frac{1}{n}\sum_{i=1}^{n}\frac{\partial U_i(\boldsymbol{\beta})}{\partial \boldsymbol{\beta}^T}\right]^{-1}\left[\frac{1}{\sqrt{n}}\sum_{i=1}^{n}\frac{\partial E(Y_i|X_i, M_i, \boldsymbol{W}_i)}{\partial \boldsymbol{\beta}}V_i^{-1}\Big(Y_i - E(Y_i|X_i, M_i, \boldsymbol{W}_i)\Big)\right] + o_p(\mathbf{1}) \\
&= -\left\{E[U_1(\boldsymbol{\beta})]\right\}^{-1}\left[\frac{1}{\sqrt{n}}\sum_{i=1}^{n}\frac{\partial E(Y_i|X_i, M_i, \boldsymbol{W}_i)}{\partial \boldsymbol{\beta}}V_i^{-1}\Big(Y_i - E(Y_i|X_i, M_i, \boldsymbol{W}_i)\Big)\right] + o_p(\mathbf{1}) \\
&= \frac{1}{\sqrt{n}}\sum_{i=1}^{n}E_{\boldsymbol{\beta}\boldsymbol{\beta}}\frac{\partial E(Y_i|X_i, M_i, \boldsymbol{W}_i)}{\partial \boldsymbol{\beta}}V_i^{-1}\Big(Y_i - E(Y_i|X_i, M_i, \boldsymbol{W}_i)\Big) + o_p(\mathbf{1}), \\
&= \frac{1}{\sqrt{n}}\sum_{i=1}^{n}\psi(Y_i, X_i, M_i, \boldsymbol{W}_i; \boldsymbol{\beta}) + o_p(\mathbf{1})
\end{aligned}$$

where $E_{\boldsymbol{\beta}\boldsymbol{\beta}} = -\left\{E[U_1(\boldsymbol{\beta})]\right\}^{-1}$. Similarly, we can expand $U(\boldsymbol{\gamma}) = 0$ around the true parameter $\boldsymbol{\gamma}$ and $U(\sigma^2) = 0$ around the true parameter σ^2 such that

$$\begin{aligned}
\sqrt{n}(\hat{\boldsymbol{\gamma}} - \boldsymbol{\gamma}) &= -\left[\frac{1}{n}\sum_{i=1}^{n}\frac{\partial U_i(\boldsymbol{\gamma})}{\partial \boldsymbol{\gamma}^T}\right]^{-1}\left[\frac{1}{\sqrt{n}}\sum_{i=1}^{n}\frac{\partial E(M_i|X_i, \boldsymbol{W}_i)}{\partial \boldsymbol{\gamma}}V_i^{*-1}\Big(M_i - E(M_i|X_i, \boldsymbol{W}_i)\Big)\right] + o_p(\mathbf{1}) \\
&= \frac{1}{\sqrt{n}}\sum_{i=1}^{n}E_{\boldsymbol{\gamma}\boldsymbol{\gamma}}\frac{\partial E(M_i|X_i, \boldsymbol{W}_i)}{\partial \boldsymbol{\gamma}}V_i^{*-1}\Big(M_i - E(M_i|X_i, \boldsymbol{W}_i)\Big) + o_p(\mathbf{1}), \\
&= \frac{1}{\sqrt{n}}\sum_{i=1}^{n}\psi(X_i, M_i, \boldsymbol{W}_i; \boldsymbol{\gamma}) + o_p(\mathbf{1}), \\
\sqrt{n}(\hat{\sigma}^2 - \sigma^2) &= -\left[\frac{1}{n}\sum_{i=1}^{n}\frac{\partial U_i(\sigma^2)}{\partial \sigma^2}\right]^{-1}\left[\frac{1}{\sqrt{n}}\sum_{i=1}^{n}\Big\{\sigma^2 - (M_i - E(M_i|X_i, \boldsymbol{W}_i))^2\Big\}\right] + o_p(\mathbf{1}) \\
&= \frac{1}{\sqrt{n}}\sum_{i=1}^{n}E_{\sigma^2\sigma^2}\Big\{\sigma^2 - (M_i - E(M_i|X_i, \boldsymbol{W}_i))^2\Big\} + o_p(\mathbf{1}), \\
&= \frac{1}{\sqrt{n}}\sum_{i=1}^{n}\psi(M_i, X_i, \boldsymbol{W}_i; \sigma^2) + o_p(\mathbf{1}),
\end{aligned}$$

where $E_{\boldsymbol{\gamma}\boldsymbol{\gamma}} = -\left\{E[U_1(\boldsymbol{\gamma})]\right\}^{-1}$ and $E_{\sigma^2\sigma^2} = -\Big\{E[U_1(\sigma^2)]\Big\}^{-1}$.

If $\text{Cov}\Big(\psi(Y, X, M, \boldsymbol{W}; \boldsymbol{\beta}), \psi_2(M, X, \boldsymbol{W}; \boldsymbol{\gamma})\Big) = 0$, then $\hat{\boldsymbol{\beta}}$ and $\hat{\boldsymbol{\gamma}}$ will be asymptotically uncorrelated. Noticing that $E[\psi(Y, X, M, \boldsymbol{W}; \boldsymbol{\beta})] = \mathbf{0}$ and $E[\psi(M, X, \boldsymbol{W}; \boldsymbol{\gamma})] = \mathbf{0}$, we have

$$\begin{aligned}
\text{Cov}\Big(\psi(Y, X, M, \boldsymbol{W}; \boldsymbol{\beta}), \psi(M, X, \boldsymbol{W}; \boldsymbol{\gamma})\Big) &= E[\psi(Y, X, M, \boldsymbol{W}; \boldsymbol{\beta})\psi^T(M, X, \boldsymbol{W}; \boldsymbol{\gamma})] \\
&= E\Big\{E\Big[\psi(Y, X, M, \boldsymbol{W}; \beta)\psi^T(M, X, \boldsymbol{W}; \boldsymbol{\gamma})|X, M, \boldsymbol{W}\Big]\Big\} \\
&= E\Big\{E[\psi(Y, X, M, \boldsymbol{W}; \boldsymbol{\beta})|X, M, \boldsymbol{W}]\psi^T(M, X, \boldsymbol{W}; \boldsymbol{\gamma})\Big\} \\
&= E\Big\{\mathbf{0} \times \psi^T(M, X, \boldsymbol{W}; \boldsymbol{\gamma})\Big\} \\
&= \mathbf{0}.
\end{aligned}$$

Thus, $\psi(Y, X, M, \boldsymbol{W}; \boldsymbol{\beta})$ and $\psi(M, X, \boldsymbol{W}; \boldsymbol{\gamma})$ are uncorrelated and therefore $\hat{\boldsymbol{\beta}}$ and $\hat{\boldsymbol{\gamma}}$ are asymptotically un-

correlated. Similarly, the covariance between $\psi(Y, X, M, \boldsymbol{W}; \boldsymbol{\beta})$ and $\psi(M, X, \boldsymbol{W}; \sigma^2)$ is

$$\begin{aligned}
\text{Cov}\Big(\psi(Y, X, M, \boldsymbol{W}; \boldsymbol{\beta}), \psi(M, X, \boldsymbol{W}; \sigma^2)\Big) &= E[\psi(Y, X, M, \boldsymbol{W}; \boldsymbol{\beta})\psi(M, X, \boldsymbol{W}; \sigma^2)] \\
&= E\Big\{E\Big[\psi(Y, X, M, \boldsymbol{W}; \beta)\psi(M, X, \boldsymbol{W}; \sigma^2)|X, M, \boldsymbol{W}\Big]\Big\} \\
&= E\Big\{E[\psi(Y, X, M, \boldsymbol{W}; \boldsymbol{\beta})|X, M, \boldsymbol{W}]\psi(M, X, \boldsymbol{W}; \sigma^2)\Big\} \\
&= E\Big\{\mathbf{0} \times \psi(M, X, \boldsymbol{W}; \sigma^2)\Big\} \\
&= \mathbf{0}.
\end{aligned}$$

It follows that $\hat{\boldsymbol{\beta}}$ and $\hat{\sigma}^2$ are asymptotically uncorrelated. In the aspect of the covariance between $\hat{\boldsymbol{\gamma}}$ and $\hat{\sigma}^2$, we have that

$$\begin{aligned}
& \text{Cov}\Big(\psi(M, X, \boldsymbol{W}; \boldsymbol{\gamma}), \psi(M, X, \boldsymbol{W}; \sigma^2)\Big) \\
= \; & E[\psi(Y, X, M, \boldsymbol{W}; \boldsymbol{\gamma})\psi(M, X, \boldsymbol{W}; \sigma^2)] \\
= \; & E\left\{E_{\boldsymbol{\gamma\gamma}} E_{\sigma^2\sigma^2} \tfrac{\partial E(M|X,\boldsymbol{W})}{\partial \boldsymbol{\gamma}} V^{*-1} \Big(M - E(M|X, \boldsymbol{W})\Big)\Big(\sigma^2 - (M - E(M|X, \boldsymbol{W}))^2\Big)\right\} \\
= \; & E_{\boldsymbol{\gamma\gamma}} E_{\sigma^2\sigma^2} \tfrac{\partial E(M|X,\boldsymbol{W})}{\partial \boldsymbol{\gamma}} V^{*-1} \left\{\sigma^2 E\left[M - E(M|X, \boldsymbol{W})\right] - E\left[M - E(M|X, \boldsymbol{W})\right]^3\right\}.
\end{aligned}$$

Obviously, $E\left[M - E(M|X, \boldsymbol{W})\right] = 0$. By noticing that M is assumed to be normally distributed in Case #3, $E\left[M - E(M|X, \boldsymbol{W})\right]^3 = 0$. It follows that

$$\begin{aligned}
\text{Cov}\Big(\psi(M, X, \boldsymbol{W}; \boldsymbol{\gamma}), \psi(M, X, \boldsymbol{W}; \sigma^2)\Big) &= E_{\boldsymbol{\gamma\gamma}} E_{\sigma^2\sigma^2} \frac{\partial E(M|X, \boldsymbol{W})}{\partial \boldsymbol{\gamma}} V^{*-1} \left\{\sigma^2 \times 0 - 0\right\} \\
&= \mathbf{0},
\end{aligned}$$

which implies $\hat{\boldsymbol{\gamma}}$ and $\hat{\sigma}^2$ are asymptotically uncorrelated. To summarize, $\hat{\boldsymbol{\beta}}$, $\hat{\boldsymbol{\gamma}}$, and $\hat{\sigma}^2$ are asymptotically uncorrelated.

Web Appendix D: Simulations in the presence of a binary confounder

We conducted additional simulation studies to examine the robustness of the product method in the presence of a binary confounder, W. This could be a representative case accounting for many confounders simultaneously. We investigate the percent bias, variance ratio, and 95% confidence interval coverage rate for Cases #1 to #4. With a focus on investigating the impact from confounding effect, in this simulation we will fix the sample size, TE, MP, and outcome prevalence while changing the confounder-exposure, confounder-mediator, and confounder-outcome associations. Specifically, we will fix the sample size at 5,000, MP to 0.5, the TE to 0.5 for the scenarios with a continuous outcome, and fix the sample size at 5,000, MP to 0.5, TE to log(1.5), and the baseline outcome prevalence at 3% for the scenarios with a binary outcome. The data generations are described as below.

First, generate W from a Bernoulli distribution with $P(W = 1) = 0.5$. Then, given W, generate a binary

exposure X based on the following logistic model

$$\text{logit}(P(X=1|W)) = \psi_0 + \psi_1 W,$$

where ψ_1 is chose to log(1.2) and log(2) such that the odds ratio of $X = 1$ for $W = 1$ versus $W = 0$ is 1.2 and 2.0 to represent a small and large confounder-exposure association, respectively. We choose ψ_0 such that the marginal probability of $X = 1$ equals 0.5. Next, we generate M from $N(\gamma_0+\gamma_1 X+\gamma_2 W, 1)$ for the scenarios with a continuous mediator (Cases #1 and #3) and from a logistic model $\text{logit}(P(M = 1|X, W)) = \gamma_0+\gamma_1 X+\gamma_2 W$ for the scenarios with a binary mediator (Cases #2 and #4). The values of γ_0 and γ_1 are selected to agree with what they were in the original simulation studies without confounder adjustment, and γ_2 is selected to 0.1 and 1 to represent a small and large confounder-mediator association, respectively. Finally, we generate the outcome Y by a normal distribution $N(\beta_0 + \beta_1 X + \beta_2 M + \beta_3 W, 1)$ for the scenarios with a continuous outcome (Cases #1 and #2) and from a logistic model $\text{logit}(P(Y = 1|X, M, W)) = \beta_0 + \beta_1 X + \beta_2 M + \beta_3 W$ for the scenarios with a binary outcome (Cases #3 and #4). The values of β_0, β_1, and β_2 is selected to agree with what they were in the original simulation studies without confounder adjustment, and β_3 are selected to 0.1 and 1 to represent a small and large confounder-outcome association, respectively.

The simulation results for the continuous outcome scenarios (Cases #1 and #2) are shown in Web Table 6 in additional file 1. We observe that both the NIE and MP estimators have minimal bias associated with accurate 95% confidence interval coverage rates among all combinations of ψ_1, γ_2, and β_3 considered. The simulation results for the binary outcome scenarios (Cases #3 and #4) are shown in Web Table 7 in additional file 1. Similar results were observed, where the percent bias of the NIE and MP estimators are controlled within 2% and the coverage rates are also close to the nominal value. All these indicate that the product method with the proposed estimation methods are robust with regard to confounding adjustment.

Web Appendix E: Causal mediation measures defined on the odds ratio scale

Here we use OR^{NIE}, OR^{NDE}, OR^{TE}, and OR^{MP} to denote the mediation measures on the odds ratio scale. The relationship between the mediation measures on the log odds ratio scale and those on the odds ratio scale is given by

$$\begin{cases} OR^{NIE} = \exp(\text{NIE}), \\ OR^{NDE} = \exp(\text{NDE}), \\ OR^{TE} = \exp(\text{TE}), \\ OR^{MP} = \dfrac{OR^{TE} - OR^{NDE}}{OR^{TE} - 1} = \dfrac{\exp(\text{TE}) - \exp(\text{NDE})}{\exp(\text{TE}) - 1}. \end{cases}$$

For NIE, NDE and TE, we can simply take exponential of point estimates and confidence interval boundaries shown in the Methodology section to construct the corresponding point and interval estimates on an odds ratio scale. In the aspect of MP, there is no one-to-one function that can map MP to OR^{MP}. However, if TE

is in small magnitude, we can show MP $\approx OR^{MP}$. Specifically, if TE ≈ 0, we can apply a first-order Taylor expansion on exp(TE) around TE $= 0$, i.e.,

$$\begin{aligned}\exp(\mathrm{TE}) - 1 &\approx \exp(\mathrm{TE})\Big|_{\mathrm{TE}=0} + \frac{\partial \exp(\mathrm{TE})}{\partial \mathrm{TE}}\Big|_{\mathrm{TE}=0} \times \mathrm{TE} - 1 \\ &= \mathrm{TE}.\end{aligned}$$

Similarly, TE ≈ 0 indicates NDE ≈ 0, therefore we also have $\exp(\mathrm{NDE}) - 1 \approx \mathrm{NDE}$. It follows that

$$OR^{MP} = \frac{\exp(\mathrm{TE}) - 1 - (\exp(\mathrm{NDE}) - 1)}{\exp(\mathrm{TE}) - 1} \approx \frac{\mathrm{TE} - \mathrm{NDE}}{\mathrm{TE}} = \mathrm{MP}.$$

References

[1] Gaynor, S. M., Schwartz, J., & Lin, X. (2019). Mediation analysis for common binary outcomes. Statistics in medicine, 38(4), 512-529.

[2] Khan, S., Spiegelman, D., Walsh, F., Mazibuko, S., Pasipamire, M., Chai, B., ... & Okello, V. (2020). Early access to antiretroviral therapy versus standard of care among HIV-positive participants in Eswatini in the public health sector: the MaxART stepped-wedge randomized controlled trial. Journal of the International AIDS Society, to appear.

[3] Liu, Q., & Pierce, D. A. (1994). A note on Gauss—Hermite quadrature. Biometrika, 81(3), 624-629.

[4] Piessens, R., de Doncker-Kapenga, E., Uberhuber, C. W., & Kahaner, D. K. (2012). Quadpack: a subroutine package for automatic integration (Vol. 1). Springer Science & Business Media.

2 Web Tables

Table 1: Simulation results for Case #2: continuous outcome and binary mediator.

			$\widehat{\text{NIE}}$				$\widehat{\text{MP}}$			
N	MP	TE	Bias(%)	CR$^{(d)}$	CR$^{(b)}$	VR	Bias(%)	CR$^{(d)}$	CR$^{(b)}$	VR
150	0.05	0.25	-25.2	**98.9**	**97.1**	1.039	-36.7	**99.1**	**98.9**	0.002
		0.5	-19.7	**96.0**	**96.2**	1.050	-18.5	**96.6**	**96.9**	0.069
		1	-12.8	**92.7**	95.2	1.042	-12.9	**92.5**	95.6	0.957
	0.2	0.25	-12.8	**92.7**	95.2	1.042	-21.6	**90.4**	**97.3**	0.000
		0.5	-7.6	**92.6**	95.3	1.043	-5.2	**92.7**	**96.8**	0.003
		1	-2.7	95.0	95.2	1.024	-2.5	**96.4**	**95.6**	0.941
	0.5	0.25	-5.9	**93.4**	95.4	1.038	-14.2	**89.6**	**97.5**	0.000
		0.5	-1.9	95.2	95.1	1.018	-1.9	**96.0**	**97.3**	0.036
		1	-0.3	94.9	94.8	1.007	-0.6	**98.1**	**95.7**	0.762
500	0.05	0.25	-3.3	**96.5**	95.1	1.013	-2.5	**98.2**	**96.9**	0.039
		0.5	-3.9	**95.7**	95.2	1.013	-3.2	**95.7**	95.3	0.937
		1	-2.6	**94.2**	95.0	1.012	-2.7	**94.1**	95.1	0.997
	0.2	0.25	-2.6	**94.2**	95.0	1.012	-0.5	**92.8**	**97.1**	0.035
		0.5	-1.6	**94.1**	95.1	1.018	-0.9	94.6	95.6	0.887
		1	-0.6	95.2	95.4	1.021	-0.5	95.3	95.2	1.004
	0.5	0.25	-1.2	94.4	95.3	1.018	0.0	**92.2**	**97.5**	0.002
		0.5	-0.4	95.5	95.4	1.024	0.0	**96.1**	95.5	0.814
		1	0.2	95.6	95.5	1.028	0.0	**96.0**	95.1	0.968
1000	0.05	0.25	-1.6	**95.6**	94.8	1.024	-2.7	**97.1**	95.2	0.733
		0.5	-2.7	95.2	95.0	1.028	-2.1	95.4	94.5	0.983
		1	-2.1	94.7	95.2	1.033	-1.6	94.4	94.8	1.019
	0.2	0.25	-2.1	94.7	95.2	1.033	0.5	**94.2**	**96.1**	0.539
		0.5	-0.8	94.5	95.0	1.031	-0.1	95.0	95.0	0.938
		1	-0.3	95.0	95.0	1.022	0.2	95.2	94.9	0.999
	0.5	0.25	-0.6	94.7	95.0	1.029	0.4	**93.8**	**97.1**	0.185
		0.5	-0.1	95.1	95.2	1.021	0.2	**95.7**	94.9	0.908
		1	0.0	94.8	94.9	1.014	0.2	95.3	95.0	0.973
5000	0.05	0.25	-0.7	95.0	95.0	0.981	-1.5	95.0	94.6	0.943
		0.5	-0.7	94.8	94.6	0.980	-0.9	94.5	**94.4**	0.972
		1	-0.3	94.8	94.8	0.978	-0.5	94.5	94.7	0.979
	0.2	0.25	-0.3	94.8	94.8	0.978	-0.6	94.6	94.9	0.909
		0.5	-0.1	95.1	94.9	0.981	-0.2	94.6	94.6	0.966
		1	-0.1	94.9	94.8	0.986	0.0	94.6	94.6	0.983
	0.5	0.25	-0.2	94.9	94.8	0.982	-0.3	95.3	95.0	0.885
		0.5	-0.1	94.9	95.0	0.988	-0.3	95.2	94.9	0.965
		1	0.0	94.9	94.9	0.991	0.0	95.1	95.0	0.982

Note: Bias(%), CR$^{(d)}$, CR$^{(b)}$, and VR denote the median percent bias, 95% confidence interval coverage rate of multivariate delta method, 95% confidence interval coverage rate of percentile bootstrap method, and mediation variance ratio, respectively. The coverage rates outside the 95% confidence boundary, i.e., $q \pm 1.96 \times \sqrt{\frac{q(1-q)}{B}}$, were highlighted in bold, where q denotes the nominal confidence interval threshold (95%) and B denotes number of replication (5,000). The median percent bias was calculated as the median of the ratio of bias to the true value over 5,000 replications, i.e., Bias(%) $= median(\frac{\hat{p}-p}{p}) \times 100\%$, where p denotes the true value of the causal mediation measure, and $\hat{p}$ is the point estimate of the simulated causal mediation measure. The median variance ratio is defined by the ratio of median delta-method variance estimators across 5,000 replications to the empirical variance of causal mediation measure estimates from the 5,000 replications.

Table 2: Simulation results for Case #3: binary outcome and continuous mediator.

				$\widehat{\text{NIE}}^{(a)}$				$\widehat{\text{NIE}}$				$\widehat{\text{MP}}^{(a)}$				$\widehat{\text{MP}}$			
N	Ncase (%)	MP	TE	Bias(%)	CR$^{(d)}$	CR$^{(b)}$	VR	Bias(%)	CR$^{(d)}$	CR$^{(b)}$	VR	Bias(%)	CR$^{(d)}$	CR$^{(b)}$	VR	Bias(%)	CR$^{(d)}$	CR$^{(b)}$	VR
	16 (3.2%)	0.05	log(1.2)	18.5	**97.3**	**93.5**	0.957	18.5	**96.8**	**93.5**	0.962	-82.2	**99.9**	**99.4**	0.000	-82.2	**99.9**	**99.4**	0.000
	18 (3.7%)	0.05	log(1.5)	-2.8	**97.3**	**93.8**	0.965	-2.8	**96.9**	**93.8**	0.968	-56.9	**99.8**	**99.1**	0.000	-56.9	**99.8**	**99.1**	0.000
	22 (4.4%)	0.05	log(2)	-5.1	**97.2**	**94.1**	0.956	-5.1	**96.8**	**94.1**	0.957	-22.2	**99.2**	**98.3**	0.000	-22.3	**99.2**	**98.3**	0.000
	16 (3.2%)	0.2	log(1.2)	-3.2	**97.5**	**93.6**	0.958	-3.2	**97.0**	**93.6**	0.962	-87.5	**92.1**	**97.6**	0.001	-87.5	**92.0**	**97.5**	0.001
500	18 (3.7%)	0.2	log(1.5)	-2.2	**96.4**	**93.7**	0.952	-2.3	**96.0**	**93.7**	0.959	-51.2	**91.3**	**97.1**	0.000	-51.4	**91.2**	**97.1**	0.000
	23 (4.6%)	0.2	log(2)	-1.0	95.2	**94.3**	0.961	-1.8	94.9	**94.3**	0.970	-11.8	**91.0**	**96.5**	0.000	-12.1	**90.9**	**96.5**	0.000
	16 (3.3%)	0.5	log(1.2)	-1.7	**96.0**	**93.3**	0.932	-1.9	**95.7**	**93.3**	0.936	-87.1	**70.8**	**93.8**	0.001	-87.1	**70.8**	**93.8**	0.001
	20 (4.1%)	0.5	log(1.5)	-0.8	**94.3**	**93.7**	0.893	-2.2	**93.9**	**93.7**	0.906	-40.4	**81.6**	**95.8**	0.000	-40.5	**81.5**	**95.8**	0.000
	31 (6.1%)	0.5	log(2)	5.4	**94.3**	**94.1**	0.889	-1.2	**93.1**	**94.0**	0.920	-3.1	**89.3**	**97.0**	0.000	-3.8	**88.9**	**96.9**	0.000
	32 (3.2%)	0.05	log(1.2)	-2.5	**96.1**	**94.1**	0.942	-2.5	**95.7**	**94.1**	0.941	-81.7	**99.7**	**99.6**	0.000	-81.7	**99.7**	**99.6**	0.000
	37 (3.7%)	0.05	log(1.5)	-8.4	**95.7**	**94.1**	0.937	-8.4	95.5	**94.1**	0.938	-33.3	**99.6**	**99.0**	0.000	-33.3	**99.6**	**99.0**	0.000
	44 (4.4%)	0.05	log(2)	-5.4	95.5	**94.0**	0.957	-5.4	95.3	**94.0**	0.957	-7.1	**98.6**	**97.1**	0.011	-7.2	**98.6**	**97.1**	0.011
	32 (3.2%)	0.2	log(1.2)	-4.6	**96.1**	**93.8**	0.958	-4.6	**95.8**	**93.8**	0.957	-78.8	**92.3**	**98.0**	0.000	-78.9	**92.3**	**98.0**	0.000
1000	37 (3.7%)	0.2	log(1.5)	-1.7	**95.7**	94.6	0.974	-1.9	95.6	94.6	0.974	-30.0	**91.3**	**97.4**	0.000	-30.1	**91.3**	**97.4**	0.000
	46 (4.6%)	0.2	log(2)	-2.5	95.1	94.7	0.985	-3.2	94.9	94.8	0.990	-5.2	**92.2**	**97.1**	0.006	-5.4	**92.1**	**97.1**	0.006
	33 (3.3%)	0.5	log(1.2)	-2.1	**95.7**	**93.9**	0.973	-2.2	95.6	**93.9**	0.973	-73.0	**73.6**	95.1	0.001	-73.0	**73.6**	95.1	0.001
	41 (4.1%)	0.5	log(1.5)	-1.2	95.0	95.4	1.011	-2.4	94.9	95.4	1.015	-19.7	**85.6**	**97.1**	0.006	-19.8	**85.5**	**97.1**	0.006
	61 (6.1%)	0.5	log(2)	5.4	95.3	95.0	0.987	-1.0	94.5	95.2	1.003	0.1	**91.6**	**97.6**	0.002	-0.6	**91.3**	**97.5**	0.002
	164 (3.2%)	0.05	log(1.2)	3.5	94.9	94.5	0.970	3.5	94.8	94.5	0.970	-29.3	**99.6**	**99.2**	0.000	-29.3	**99.6**	**99.2**	0.000
	186 (3.7%)	0.05	log(1.5)	-1.8	95.2	94.9	0.996	-1.8	95.1	94.9	0.996	-4.3	**98.4**	**96.6**	0.035	-4.3	**98.4**	**96.6**	0.035
	221 (4.4%)	0.05	log(2)	0.3	94.9	94.5	0.981	0.3	94.8	94.5	0.981	-0.4	**96.6**	95.0	0.864	-0.4	**96.6**	95.0	0.864
	165 (3.2%)	0.2	log(1.2)	1.0	94.8	94.6	0.984	1.0	94.8	94.6	0.984	-27.7	**92.5**	**97.6**	0.000	-27.7	**92.5**	**97.6**	0.000
5000	189 (3.7%)	0.2	log(1.5)	0.9	94.7	94.7	0.991	0.7	94.7	94.7	0.991	-0.6	**93.0**	**97.0**	0.003	-0.6	**93.0**	**97.0**	0.003
	232 (4.6%)	0.2	log(2)	0.5	95.2	95.0	1.039	-0.1	95.1	95.0	1.039	0.4	94.5	95.3	0.777	0.3	94.4	95.3	0.776
	168 (3.3%)	0.5	log(1.2)	0.2	94.8	94.8	0.979	0.0	94.8	94.7	0.980	-21.7	**84.3**	**96.6**	0.000	-21.7	**84.3**	**96.6**	0.000
	207 (4.1%)	0.5	log(1.5)	1.0	95.1	95.3	1.015	-0.2	95.1	95.3	1.015	0.0	**91.4**	**97.0**	0.001	-0.1	**91.3**	**97.0**	0.001
	309 (6.1%)	0.5	log(2)	6.3	**92.5**	**91.0**	0.999	0.0	95.2	95.3	1.005	0.7	94.6	94.5	0.767	0.0	**94.0**	**94.4**	0.766
	657 (3.2%)	0.05	log(1.2)	2.5	95.3	\	1.024	2.5	95.3	\	1.024	0.0	**98.5**	\	0.002	0.0	**98.5**	\	0.002
	744 (3.7%)	0.05	log(1.5)	-0.3	95.5	\	1.017	-0.3	95.5	\	1.017	0.2	**96.6**	\	0.903	0.2	**96.6**	\	0.903
	885 (4.4%)	0.05	log(2)	0.1	95.2	\	1.018	0.1	95.2	\	1.018	0.5	95.4	\	0.982	0.5	95.4	\	0.982
	660 (3.2%)	0.2	log(1.2)	0.6	95.2	\	1.010	0.6	95.2	\	1.010	-0.7	**92.9**	\	0.000	-0.7	**92.9**	\	0.000
20000	756 (3.7%)	0.2	log(1.5)	0.0	95.3	\	1.004	-0.2	95.2	\	1.004	0.3	94.6	\	0.783	0.3	94.6	\	0.783
	929 (4.6%)	0.2	log(2)	0.6	94.9	\	1.008	0.0	94.9	\	1.009	0.2	95.0	\	0.947	0.1	95.0	\	0.947
	672 (3.3%)	0.5	log(1.2)	0.4	95.4	\	1.007	0.3	95.3	\	1.007	-1.1	**90.8**	\	0.000	-1.1	**90.8**	\	0.000
	831 (4.1%)	0.5	log(1.5)	1.1	95.1	\	1.013	-0.1	95.3	\	1.013	0.4	**94.3**	\	0.781	0.3	**94.2**	\	0.781
	1237 (6.1%)	0.5	log(2)	6.1	**79.5**	\	0.980	-0.1	94.7	\	0.980	0.9	95.0	\	0.945	0.2	94.7	\	0.944

Note: Ncase, Bias(%), CR$^{(d)}$, CR$^{(b)}$, and VR denote the average number of cases under each setting across 5,000 replications, the median percent bias, 95% confidence interval coverage rate of multivariate delta method, 95% confidence interval coverage rate of percentile bootstrap method, and mediation variance ratio, respectively. The coverage rates outside the 95% confidence boundary, i.e., $q \pm 1.96 \times \sqrt{\frac{q(1-q)}{B}}$, were highlighted in bold, where q denotes the nominal confidence interval threshold (95%) and B denotes number of replication (5,000). The median percent bias was calculated as the median of the ratio of bias to the true value over 5,000 replications, i.e., Bias(%) $= median(\frac{\hat{p}-p}{p}) \times 100\%$, where p denotes the true value of the causal mediation measure, and $\hat{p}$ is the point estimate of the simulated causal mediation measure. The median variance ratio is defined by the ratio of median delta-method variance estimators across 5,000 replications to the empirical variance of causal mediation measure estimates from the 5,000 replications.

Table 3: Simulation results for Case #4: binary outcome and binary mediator.

				$\widehat{\text{NIE}}^{(a)}$				$\widehat{\text{NIE}}$				$\widehat{\text{MP}}^{(a)}$				$\widehat{\text{MP}}$			
N	Ncase (%)	MP	TE	Bias(%)	CR$^{(d)}$	CR$^{(b)}$	VR	Bias(%)	CR$^{(d)}$	CR$^{(b)}$	VR	Bias(%)	CR$^{(d)}$	CR$^{(b)}$	VR	Bias(%)	CR$^{(d)}$	CR$^{(b)}$	VR
	16 (3.3%)	0.05	log(1.2)	-50.0	**93.1**	**93.7**	0.867	-50.0	**93.4**	**93.7**	0.866	-92.1	**99.5**	**99.4**	0.000	-91.6	**99.5**	**99.4**	0.000
	18 (3.7%)	0.05	log(1.5)	-17.7	**93.9**	**94.1**	0.892	-17.8	**94.4**	**94.1**	0.893	-61.3	**99.2**	**99.3**	0.000	-61.2	**99.3**	**99.3**	0.000
	22 (4.6%)	0.05	log(2)	-6.0	**94.3**	**94.1**	0.899	-6.4	94.7	**94.1**	0.903	-22.9	**98.1**	**98.3**	0.000	-23.3	**98.3**	**98.3**	0.000
	17 (3.4%)	0.2	log(1.2)	-10.0	**92.8**	**93.8**	0.868	-10.3	**93.3**	**93.8**	0.868	-88.8	**89.9**	**97.5**	0.000	-88.7	**90.1**	**97.5**	0.000
500	20 (4.0%)	0.2	log(1.5)	-3.5	**93.2**	94.5	0.912	-4.2	**93.5**	94.5	0.908	-50.5	**90.3**	**97.3**	0.000	-50.8	**90.2**	**97.3**	0.000
	26 (5.2%)	0.2	log(2)	-2.8	**94.0**	94.9	0.940	-4.0	**94.1**	94.9	0.935	-13.9	**90.1**	**96.8**	0.000	-14.5	**90.0**	**96.8**	0.000
	18 (3.6%)	0.5	log(1.2)	-5.5	**92.6**	**94.3**	0.908	-6.0	**92.9**	**94.3**	0.908	-84.8	**70.8**	**94.2**	0.000	-84.7	**70.9**	**94.3**	0.000
	24 (4.8%)	0.5	log(1.5)	-1.7	**93.1**	94.9	0.949	-3.0	**93.1**	94.9	0.942	-37.0	**81.9**	**95.7**	0.000	-37.1	**81.9**	**95.7**	0.000
	37 (7.5%)	0.5	log(2)	-0.5	**93.9**	95.4	0.998	-1.5	**93.5**	95.1	0.991	-6.1	**88.7**	**96.6**	0.000	-3.4	**89.7**	**97.0**	0.000
	33 (3.3%)	0.05	log(1.2)	-7.5	**94.2**	94.9	0.922	-7.5	94.4	94.9	0.922	-89.5	**99.4**	**99.4**	0.000	-89.3	**99.5**	**99.4**	0.000
	37 (3.7%)	0.05	log(1.5)	-2.5	**94.3**	94.7	0.935	-2.7	94.5	94.7	0.933	-32.6	**98.8**	**98.7**	0.000	-32.7	**98.8**	**98.6**	0.000
	23 (4.6%)	0.05	log(2)	-4.4	**94.4**	94.7	0.959	-4.9	94.7	94.6	0.961	-5.5	**97.3**	**97.0**	0.010	-6.0	**97.6**	**97.0**	0.010
	34 (3.4%)	0.2	log(1.2)	0.8	**94.3**	94.6	0.936	0.5	94.4	94.6	0.933	-78.4	**91.4**	**97.9**	0.000	-78.4	**91.5**	**97.8**	0.000
1000	40 (4.0%)	0.2	log(1.5)	-1.5	**94.0**	94.7	0.941	-2.2	**94.2**	94.6	0.937	-25.8	**90.9**	**97.2**	0.000	-26.2	**90.8**	**97.2**	0.000
	52 (5.2%)	0.2	log(2)	-0.7	**94.2**	94.8	0.957	-2.1	**94.2**	94.9	0.956	-1.8	**91.6**	**96.6**	0.005	-2.4	**91.5**	**96.5**	0.005
	36 (3.6%)	0.5	log(1.2)	-0.7	**94.4**	95.0	0.944	-1.3	94.5	95.0	0.942	-70.8	**75.6**	95.5	0.000	-70.9	**75.6**	95.5	0.000
	48 (4.8%)	0.5	log(1.5)	0.4	**94.2**	95.1	0.970	-0.8	**94.3**	95.2	0.968	-14.0	**87.1**	**97.2**	0.000	-14.0	**87.0**	**97.2**	0.000
	75 (7.5%)	0.5	log(2)	0.4	94.8	95.2	0.990	-1.0	94.5	95.1	0.984	-2.8	**90.4**	**96.7**	0.004	-0.7	**91.3**	**97.0**	0.007
	166 (3.3%)	0.05	log(1.2)	-14.5	95.1	95.2	0.995	-24.5	95.2	95.2	0.995	-42.4	**99.3**	**98.2**	0.002	-42.5	**99.4**	**99.2**	0.001
	190 (3.7%)	0.05	log(1.5)	-3.4	94.9	95.1	0.996	-3.6	95.0	95.1	0.995	-4.7	**97.7**	**96.9**	0.011	-4.8	**97.7**	**96.9**	0.015
	229 (4.6%)	0.05	log(2)	-2.2	94.9	95.0	0.998	-2.6	95.0	95.1	0.999	-1.6	**96.3**	95.4	0.890	-2.0	**96.3**	95.4	0.891
	171 (3.4%)	0.2	log(1.2)	-4.1	94.8	95.1	0.991	-4.4	94.9	95.1	0.990	-31.3	**92.4**	**97.8**	0.001	-31.4	**92.3**	**98.0**	0.000
5000	204 (4.0%)	0.2	log(1.5)	-0.9	95.1	95.2	0.999	-1.6	95.0	95.3	1.000	-1.2	**93.5**	**97.5**	0.049	-1.7	**93.3**	**97.4**	0.053
	261 (5.2%)	0.2	log(2)	0.5	95.0	95.1	0.991	-0.9	95.0	95.2	0.990	0.4	95.2	95.3	0.832	-0.3	94.9	95.0	0.829
	183 (3.6%)	0.5	log(1.2)	-1.4	94.8	95.1	0.980	-2.0	94.7	95.0	0.980	-21.8	**85.5**	**97.4**	0.000	-22.1	**85.4**	**97.4**	0.000
	242 (4.8%)	0.5	log(1.5)	0.2	94.9	95.0	0.981	-1.0	94.7	94.9	0.980	0.5	**92.1**	**97.6**	0.000	0.4	**92.0**	**97.6**	0.000
	378 (7.5%)	0.5	log(2)	1.0	94.7	94.7	0.979	-0.3	94.6	94.6	0.979	-1.9	**93.7**	95.1	0.851	-0.1	94.9	95.3	0.861
	663 (3.3%)	0.05	log(1.2)	-5.1	95.1	\	1.022	-5.2	95.1	\	1.022	-7.6	**98.4**	\	0.000	-7.7	**98.4**	\	0.000
	759 (3.7%)	0.05	log(1.5)	-1.2	94.8	\	1.008	-1.4	94.8	\	1.008	-1.5	**96.3**	\	0.886	-1.7	**96.3**	\	0.888
	916 (4.6%)	0.05	log(2)	-0.1	95.3	\	1.016	-0.5	95.2	\	1.015	-0.5	**95.8**	\	0.973	-0.9	**95.7**	\	0.973
	684 (3.4%)	0.2	log(1.2)	-1.1	94.6	\	0.994	-1.4	94.6	\	0.993	-2.0	**93.1**	\	0.002	-2.2	**93.1**	\	0.002
20000	815 (4.0%)	0.2	log(1.5)	0.5	94.7	\	1.007	-0.1	94.6	\	1.007	0.2	95.1	\	0.793	-0.2	94.8	\	0.792
	1043 (5.2%)	0.2	log(2)	0.9	95.1	\	0.999	-0.5	95.1	\	0.999	0.3	95.2	\	0.931	-0.4	95.0	\	0.931
	731 (3.6%)	0.5	log(1.2)	0.3	94.7	\	0.996	-0.3	94.7	\	0.995	-0.7	**91.0**	\	0.000	-1.0	**90.9**	\	0.000
	969 (4.8%)	0.5	log(1.5)	1.0	94.8	\	0.994	-0.2	94.8	\	0.994	0.1	**94.3**	\	0.796	0.0	**94.3**	\	0.797
	1514 (7.5%)	0.5	log(2)	1.1	94.7	\	0.995	-0.1	94.9	\	0.995	-1.7	**92.7**	\	0.927	0.0	94.5	\	0.930

Note: Ncase, Bias(%), CR$^{(d)}$, CR$^{(b)}$, and VR denote the average number of cases under each setting across 5,000 replications, the median percent bias, 95% confidence interval coverage rate of multivariate delta method, 95% confidence interval coverage rate of percentile bootstrap method, and mediation variance ratio, respectively. The coverage rates outside the 95% confidence boundary, i.e., $q \pm 1.96 \times \sqrt{\frac{q(1-q)}{B}}$, were highlighted in bold, where q denotes the nominal confidence interval threshold (95%) and B denotes number of replication (5,000). The median percent bias was calculated as the median of the ratio of bias to the true value over 5,000 replications, i.e., Bias(%) $= median(\frac{\hat{p}-p}{p}) \times 100\%$, where p denotes the true value of the causal mediation measure, and $\hat{p}$ is the point estimate of the simulated causal mediation measure. The median variance ratio is defined by the ratio of median delta-method variance estimators across 5,000 replications to the empirical variance of causal mediation measure estimates from the 5,000 replications.

Table 4: Sensitivity analysis for the homoskedasticity assumption in Case #3. Here, we set $\text{Var}(M|X) = \eta_0 + \eta_1 X$, where $\eta_1 = 0, 0.25, 0.5, 0.75, 1$ to represent increasingly levels of heteroskedasticity and η_0 is set to restrict the expectation of $\text{Var}(M|X)$ to equal 1. Simulation results for $\eta = 0$ indicates homoskedasticity and are added as benchmark.

					$\widehat{\text{NIE}}^{(a)}$			$\widehat{\text{NIE}}$			$\widehat{\text{MP}}^{(a)}$			$\widehat{\text{MP}}$		
η_1	Prev	MP	TE	Ncase(%)	Bias(%)	CR$^{(d)}$	VR	Bias(%)	CR$^{(d)}$	VR	Bias(%)	CR$^{(d)}$	VR	Bias(%)	CR$^{(d)}$	VR
0	0.03	0.1	log(1.2)	659 (3.3%)	1.2	95.48	1.016	1.2	95.4	1.016	-0.8	**96.6**	0.000	-0.8	**96.6**	0.000
			log(2)	892 (4.4%)	0.3	95.02	1.004	0.1	95.0	1.004	0.3	94.7	0.956	0.3	94.7	0.956
		0.5	log(1.2)	672 (3.4%)	0.4	95.38	1.007	0.3	95.3	1.007	-1.1	**90.8**	0.000	-1.1	**90.8**	0.000
			log(2)	1235 (6.2%)	6.1	**79.54**	0.980	-0.1	94.7	0.980	0.9	95.0	0.945	0.2	94.7	0.944
	0.5	0.1	log(1.2)	10455 (52.3%)	-0.1	95.18	1.013	-0.1	95.2	1.012	0.0	**96.0**	0.913	0.0	**96.0**	0.913
			log(2)	11671 (58.4%)	0.7	94.82	0.985	0.1	94.8	0.985	-0.1	95.0	0.974	0.0	95.0	0.974
		0.5	log(1.2)	10450 (52.3%)	1.2	95.06	0.996	-0.1	95.1	0.997	-0.1	**93.9**	0.819	-0.1	**93.9**	0.819
			log(2)	11664 (58.3%)	21.0	**0.58**	0.980	0.0	95.0	0.984	-0.4	**93.8**	0.967	-0.1	94.4	0.967
0.25	0.03	0.1	log(1.2)	658 (3.3%)	1.5	95.36	1.014	1.5	95.4	1.014	-1.0	**96.5**	0.002	-1.0	**96.5**	0.002
			log(2)	898 (4.5%)	0.3	94.86	0.996	0.2	94.8	0.996	0.3	94.8	0.947	0.3	94.9	0.947
		0.5	log(1.2)	675 (3.4%)	0.2	94.96	1.001	0.1	95.0	1.000	-1.0	**90.7**	0.004	-1.0	**90.7**	0.004
			log(2)	1245 (6.2%)	6.2	**78.84**	0.988	-0.1	94.6	0.987	0.7	95.1	0.950	0.1	94.8	0.949
	0.5	0.1	log(1.2)	10446 (52.2%)	-0.1	94.94	1.011	-0.1	94.9	1.011	0.0	**95.7**	0.913	0.0	**95.7**	0.913
			log(2)	11666 (58.3%)	0.6	94.66	0.983	-0.1	94.7	0.983	-0.2	95.1	0.975	-0.2	95.1	0.975
		0.5	log(1.2)	10435 (52.1%)	1.3	95.10	0.985	0.0	95.0	0.985	0.1	**93.9**	0.824	0.1	**93.9**	0.824
			log(2)	11641 (58.2%)	21.0	**0.54**	0.983	0.0	95.0	0.986	-0.4	**94.2**	0.977	0.0	94.5	0.977
0.5	0.03	0.1	log(1.2)	657 (3.3%)	1.2	95.48	1.011	1.2	95.5	1.011	-1.2	**96.5**	0.001	-1.2	**96.5**	0.001
			log(2)	901 (4.5%)	0.3	94.72	0.991	0.2	94.7	0.991	0.0	94.8	0.939	0.0	94.8	0.938
		0.5	log(1.2)	670 (3.3%)	0.1	95.06	1.001	0.0	95.0	1.001	-1.6	**90.6**	0.000	-1.6	**90.6**	0.000
			log(2)	1261 (6.3%)	6.1	**79.14**	0.989	-0.1	95.0	0.989	0.8	95.3	0.948	0.1	94.8	0.948
	0.5	0.1	log(1.2)	10472 (52.3%)	0.0	95.06	1.009	0.0	95.1	1.009	-0.1	**95.6**	0.913	-0.1	**95.6**	0.913
			log(2)	11648 (58.2%)	0.7	94.94	0.986	0.1	94.7	0.985	-0.2	94.9	0.977	-0.1	94.9	0.977
		0.5	log(1.2)	10448 (52.2%)	1.2	95.04	0.979	-0.1	94.9	0.979	-0.1	**94.0**	0.818	-0.1	**94.0**	0.818
			log(2)	11622 (58.1%)	21.0	**0.48**	0.980	-0.1	94.9	0.983	-0.3	**94.1**	0.973	0.0	94.4	0.973
0.75	0.03	0.1	log(1.2)	655 (3.2%)	1.0	**95.64**	1.006	1.0	**95.6**	1.006	-2.6	**96.5**	0.005	-2.6	**96.5**	0.005
			log(2)	897 (4.4%)	0.7	94.50	0.984	0.6	94.5	0.984	0.2	94.9	0.932	0.2	94.8	0.931
		0.5	log(1.2)	670 (3.3%)	0.4	94.72	0.992	0.2	94.7	0.992	-1.2	**90.5**	0.001	-1.2	**90.5**	0.001
			log(2)	1263 (6.3%)	6.1	**79.52**	0.983	-0.1	94.7	0.983	0.7	95.1	0.935	0.1	94.7	0.935
	0.5	0.1	log(1.2)	10451 (52.2%)	-0.1	95.30	1.007	-0.2	95.3	1.006	-0.4	95.6	0.912	-0.4	95.6	0.912
			log(2)	11673 (58.3%)	0.7	94.82	0.983	0.0	94.8	0.984	-0.2	94.6	0.977	-0.1	94.6	0.977
		0.5	log(1.2)	10433 (52.1%)	1.1	94.94	0.976	-0.1	94.9	0.977	-0.1	**94.0**	0.814	-0.1	**94.0**	0.814
			log(2)	11593 (57.9%)	20.9	**0.56**	0.981	-0.1	94.9	0.983	-0.3	**94.0**	0.967	0.1	**94.2**	0.967
1	0.03	0.1	log(1.2)	654 (3.2%)	1.0	**95.70**	1.004	1.0	**95.7**	1.004	-2.5	**96.4**	0.000	-2.5	**96.4**	0.000
			log(2)	896 (4.4%)	0.5	94.72	0.981	0.3	94.7	0.981	0.2	94.9	0.929	0.2	94.9	0.928
		0.5	log(1.2)	670 (3.3%)	0.3	94.72	0.987	0.2	94.7	0.987	-1.3	**90.4**	0.000	-1.3	**90.4**	0.000
			log(2)	1263 (6.3%)	6.2	**79.08**	0.990	0.0	94.8	0.989	1.0	95.3	0.938	0.3	94.7	0.938
	0.5	0.1	log(1.2)	10448 (52.2%)	-0.3	95.26	1.006	-0.4	95.3	1.006	-0.5	95.4	0.912	-0.5	95.4	0.912
			log(2)	11667 (58.3%)	0.7	94.70	0.980	0.1	94.8	0.980	-0.3	94.5	0.975	-0.2	94.5	0.975
		0.5	log(1.2)	10425 (52.1%)	1.1	94.98	0.975	-0.1	95.0	0.974	-0.1	**93.9**	0.817	-0.1	**93.9**	0.817
			log(2)	11580 (57.9%)	20.9	**0.48**	0.982	-0.1	94.9	0.982	-0.4	**93.9**	0.963	0.0	**94.2**	0.964

Note: Prev, Ncase, Bias(%), CR$^{(d)}$, and VR denote the baseline outcome prevalence, the average number of cases under each setting across 5,000 replications, the median percent bias, 95% confidence interval coverage rate of multivariate delta method, and mediation variance ratio, respectively. The coverage rates outside the 95% confidence boundary, i.e., $q \pm 1.96 \times \sqrt{\frac{q(1-q)}{B}}$, were highlighted in bold, where q denotes the nominal confidence interval threshold (95%) and B denotes number of replication (5,000). The median percent bias was calculated as the median of the ratio of bias to the true value over 5,000 replications, i.e., $\text{Bias}(\%) = median(\frac{\hat{p}-p}{p}) \times 100\%$, where p is the true value of the causal mediation measure evaluated based on equations (5) and (6) in manuscript, and $\hat{p}$ is the point estimate of the simulated causal mediation measure. The median variance ratio is defined by the ratio of median delta-method variance estimators across 5,000 replications to the empirical variance of causal mediation measure estimates from the 5,000 replications.

Table 5: Sensitivity analysis for the normality assumption in Case #3. Here, $M|X$ was set as a gamma distribution with coefficient of skewness at 1, 1.5 and 2. Simulation results when $M|X$ follows normality were added as benchmark (upper panel with Skewness = 0).

					$\widehat{\text{NIE}}^{(a)}$			$\widehat{\text{NIE}}$			$\widehat{\text{MP}}^{(a)}$			$\widehat{\text{MP}}$		
Skewness	Prev	MP	TE	Ncase(%)	Bias(%)	CR$^{(d)}$	VR	Bias(%)	CR$^{(d)}$	VR	Bias(%)	CR$^{(d)}$	VR	Bias(%)	CR$^{(d)}$	VR
0	0.03	0.1	log(1.2)	659 (3.3%)	1.2	95.48	1.016	1.2	95.4	1.016	-0.8	**96.6**	0.000	-0.8	**96.6**	0.000
			log(2)	892 (4.4%)	0.3	95.02	1.004	0.1	95.0	1.004	0.3	94.7	0.956	0.3	94.7	0.956
		0.5	log(1.2)	672 (3.4%)	0.4	95.38	1.007	0.3	95.3	1.007	-1.1	**90.8**	0.000	-1.1	**90.8**	0.000
			log(2)	1235 (6.2%)	6.1	**79.54**	0.980	-0.1	94.7	0.980	0.9	95.0	0.945	0.2	94.7	0.944
	0.5	0.1	log(1.2)	10455 (52.3%)	-0.1	95.18	1.013	-0.1	95.2	1.012	0.0	**96.0**	0.913	0.0	**96.0**	0.913
			log(2)	11671 (58.4%)	0.7	94.82	0.985	0.1	94.8	0.985	-0.1	95.0	0.974	0.0	95.0	0.974
		0.5	log(1.2)	10450 (52.3%)	1.2	95.06	0.996	-0.1	95.1	0.997	-0.1	**93.9**	0.819	-0.1	**93.9**	0.819
			log(2)	11664 (58.3%)	21.0	**0.58**	0.980	0.0	95.0	0.984	-0.4	**93.8**	0.967	-0.1	94.4	0.967
1	0.03	0.1	log(1.2)	659 (3.3%)	-1.7	94.86	0.995	-1.7	94.9	0.995	-3.8	**96.7**	0.001	-3.8	**96.7**	0.001
			log(2)	896 (4.5%)	-0.3	94.90	0.983	-0.4	94.9	0.983	-0.3	95.5	0.950	-0.3	95.5	0.950
		0.5	log(1.2)	674 (3.4%)	-0.2	94.78	0.980	-0.3	94.7	0.981	-1.2	**90.7**	0.000	-1.3	**90.7**	0.000
			log(2)	1287 (6.4%)	6.1	**76.28**	1.000	-0.1	95.1	1.002	0.9	**95.9**	0.965	0.3	95.6	0.965
	0.5	0.1	log(1.2)	10456 (52.3%)	1.0	94.84	0.975	0.9	94.8	0.975	0.9	95.6	0.881	0.9	95.6	0.881
			log(2)	11661 (58.3%)	0.7	94.50	0.981	0.0	94.5	0.981	0.1	94.5	0.975	0.2	94.5	0.975
		0.5	log(1.2)	10453 (52.3%)	1.3	94.66	0.994	0.0	95.1	0.994	0.2	95.4	0.795	0.2	95.4	0.795
			log(2)	11507 (57.6%)	21.0	**0.64**	0.992	-0.1	94.9	0.997	-0.3	95.0	1.001	0.1	95.2	1.001
1.5	0.03	0.1	log(1.2)	658 (3.3%)	-1.6	95.28	0.999	-1.6	95.3	0.999	-4.0	**96.5**	0.000	-4.0	**96.5**	0.000
			log(2)	895 (4.5%)	0.2	95.16	1.012	0.0	95.2	1.012	-0.2	**95.6**	0.963	-0.2	**95.6**	0.963
		0.5	log(1.2)	674 (3.4%)	0.0	95.14	1.005	-0.2	95.1	1.005	-1.5	**90.0**	0.002	-1.5	**90.0**	0.002
			log(2)	1303 (6.5%)	6.2	**74.16**	0.986	-0.1	95.1	0.984	0.7	95.2	0.917	0.0	94.8	0.917
	0.5	0.1	log(1.2)	10453 (52.3%)	0.1	94.98	1.001	0.0	95.0	1.001	0.3	**96.5**	0.912	0.3	**96.5**	0.912
			log(2)	11663 (58.3%)	0.8	94.94	0.999	0.1	94.9	0.999	0.1	95.2	0.991	0.2	95.3	0.991
		0.5	log(1.2)	10449 (52.2%)	1.3	94.86	1.018	0.0	95.0	1.018	0.3	95.1	0.825	0.3	95.1	0.825
			log(2)	11462 (57.3%)	21.0	**0.58**	1.023	0.0	95.0	1.020	-0.3	95.1	0.999	0.0	95.4	0.999
2	0.03	0.1	log(1.2)	660 (3.3%)	-2.9	95.14	0.998	-2.9	95.1	0.998	-3.7	**97.2**	0.000	-3.7	**97.2**	0.000
			log(2)	894 (4.4%)	-0.2	95.52	1.004	-0.3	95.5	1.004	-0.3	95.2	0.962	-0.3	95.3	0.962
		0.5	log(1.2)	675 (3.4%)	-0.1	94.76	0.982	-0.2	94.7	0.982	0.1	**90.8**	0.000	0.1	**90.8**	0.000
			log(2)	1311 (6.5%)	6.1	**73.38**	1.000	-0.1	94.6	1.000	0.7	**95.7**	0.949	0.0	95.3	0.950
	0.5	0.1	log(1.2)	10459 (52.3%)	-0.7	95.22	0.993	-0.7	95.2	0.994	-0.5	**95.9**	0.873	-0.5	**95.9**	0.873
			log(2)	11668 (58.3%)	0.4	95.44	0.995	-0.3	95.3	0.995	-0.3	94.7	0.969	-0.2	94.7	0.969
		0.5	log(1.2)	10441 (52.2%)	0.9	94.88	0.991	-0.3	94.8	0.991	-0.2	94.6	0.799	-0.2	94.6	0.799
			log(2)	11426 (57.1%)	21.0	**0.96**	0.988	-0.1	94.9	0.992	-0.4	94.4	0.950	-0.1	94.4	0.950

Note: Prev, Ncase, Bias(%), CR$^{(d)}$, and VR denote the baseline outcome prevalence, the average number of cases under each setting across 5,000 replications, the median percent bias, 95% confidence interval coverage rate of multivariate delta method, and mediation variance ratio, respectively. The coverage rates outside the 95% confidence boundary, i.e., $q \pm 1.96 \times \sqrt{\frac{q(1-q)}{B}}$, were highlighted in bold, where q denotes the nominal confidence interval threshold (95%) and B denotes number of replication (5,000). The median percent bias was calculated as the median of the ratio of bias to the true value over 5,000 replications, i.e., Bias(%) $= median(\frac{\hat{p}-p}{p}) \times 100\%$, where p is the true value of the causal mediation measure evaluated based on equations (5) and (6) in manuscript, and $\hat{p}$ is the point estimate of the simulated causal mediation measure. The median variance ratio is defined by the ratio of median delta-method variance estimators across 5,000 replications to the empirical variance of causal mediation measure estimates from the 5,000 replications.

Table 6: Simulation results in the presence of a binary confounder for the scenarios with a continuous outcome (Cases #1 and Cases #2)

			$\widehat{\text{NIE}}$			$\widehat{\text{MP}}$		
ψ_1	γ_2	β_3	Bias(%)	CR$^{(d)}$	VR	Bias(%)	CR$^{(d)}$	VR
Case #1: continuous outcome and continuous mediator								
log(1.2)	0.1	0.1	0.2	**95.8**	1.035	0.2	95.1	0.968
		1	-0.1	95.0	0.994	0.2	95.1	1.014
	1	0.1	0.1	95.0	0.988	-0.1	94.9	0.946
		1	0.0	95.2	1.012	-0.2	**95.7**	1.014
log(2)	0.1	0.1	-0.1	94.9	0.979	-0.1	94.8	0.963
		1	-0.4	95.0	0.991	0.1	95.1	0.973
	1	0.1	0.0	95.3	0.992	-0.3	94.8	0.965
		1	-0.2	95.1	1.026	0.0	95.3	0.995
Case #2: continuous outcome and binary mediator								
log(1.2)	0.1	0.1	-0.1	94.8	0.971	-0.1	94.9	0.978
		1	0.0	94.9	1.002	0.1	95.5	1.003
	1	0.1	0.0	95.1	1.006	-0.1	95.1	0.969
		1	-0.1	94.9	1.017	0.0	95.2	0.994
log(2)	0.1	0.1	0.1	95.2	1.007	0.1	95.0	0.976
		1	-0.1	94.4	0.980	0.0	95.2	0.979
	1	0.1	-0.3	95.3	1.031	0.1	95.3	1.008
		1	0.0	94.4	0.965	0.0	94.9	0.965

Note: ψ_1, γ_2 and β_3 represent the strengthness of the confounder-exposure, confounder-mediator, and confounder-outcome associations. Bias(%), CR$^{(d)}$, and VR denote the median percent bias, 95% confidence interval coverage rate of multivariate delta method, and mediation variance ratio, respectively. The coverage rates outside the 95% confidence boundary, i.e., $q \pm 1.96 \times \sqrt{\frac{q(1-q)}{B}}$, were highlighted in bold, where q denotes the nominal confidence interval threshold (95%) and B denotes number of replication (5,000). The median percent bias was calculated as the median of the ratio of bias to the true value over 5,000 replications, i.e., Bias(%) $= median(\frac{\hat{p}-p}{p}) \times 100\%$, where p denotes the true value of the causal mediation measure, and $\hat{p}$ is the point estimate of the simulated causal mediation measure. The median variance ratio is defined by the ratio of median delta-method variance estimators across 5,000 replications to the empirical variance of causal mediation measure estimates from the 5,000 replications.

Table 7: Simulation results in the presence of a binary confounder for the scenarios with a binary outcome (Cases #3 and Cases #4)

			$\widehat{\text{NIE}}^{(a)}$			$\widehat{\text{NIE}}$			$\widehat{\text{MP}}^{(a)}$			$\widehat{\text{MP}}$		
ψ_1	γ_2	β_3	Bias(%)	CR$^{(d)}$	VR	Bias(%)	CR$^{(d)}$	VR	Bias(%)	CR$^{(d)}$	VR	Bias(%)	CR$^{(d)}$	VR
Case #3: binary outcome and continuous mediator														
log(1.2)	0.1	0.1	1.0	94.9	0.991	-0.2	94.5	0.992	0.0	**92.0**	0.006	-0.1	**91.9**	0.006
		1	0.7	94.9	0.987	-0.4	95.0	0.988	0.5	**92.8**	0.131	0.4	**92.7**	0.131
	1	0.1	1.1	95.4	1.027	-0.1	95.3	1.029	0.5	**92.2**	0.027	0.4	**92.2**	0.027
		1	1.0	94.5	0.984	-0.2	94.4	0.984	0.0	**93.2**	0.486	-0.1	**93.2**	0.486
log(2)	0.1	0.1	0.6	94.8	0.942	-0.6	94.5	0.944	-0.9	**91.0**	0.055	-1.0	**90.9**	0.055
		1	1.1	95.0	0.995	-0.1	94.6	0.995	-0.5	**92.6**	0.212	-0.6	**92.6**	0.211
	1	0.1	1.1	94.8	0.983	-0.1	94.7	0.984	0.4	**91.9**	0.000	0.3	**91.9**	0.000
		1	1.0	95.1	1.010	-0.2	94.8	1.011	0.2	**92.7**	0.512	0.1	**92.6**	0.512
Case #4: binary outcome and binary mediator														
log(1.2)	0.1	0.1	0.6	94.9	0.996	-0.6	94.7	0.996	-0.8	**91.1**	0.003	-0.9	**91.1**	0.001
		1	1.1	94.9	0.985	-0.2	94.7	0.985	0.1	**92.7**	0.476	0.0	**92.7**	0.478
	1	0.1	1.0	95.5	1.014	-0.2	95.1	1.014	-0.2	**92.0**	0.005	-0.3	**91.9**	0.003
		1	0.7	94.6	0.977	-0.6	94.6	0.976	-0.1	**93.3**	0.176	-0.2	**93.2**	0.184
log(2)	0.1	0.1	1.4	94.5	0.944	0.2	**94.4**	0.943	0.6	**91.8**	0.000	0.5	**91.8**	0.002
		1	1.0	94.9	1.015	-0.2	94.8	1.014	0.1	**93.0**	0.512	0.0	**93.0**	0.514
	1	0.1	1.1	94.7	0.990	-0.1	**94.4**	0.989	-0.1	**92.2**	0.035	-0.1	**92.2**	0.072
		1	1.2	94.7	1.009	0.0	94.8	1.010	0.7	**93.4**	0.487	0.6	**93.4**	0.490

Note: ψ_1, γ_2 and β_3 represent the strengthness of the confounder-exposure, confounder-mediator, and confounder-outcome associations. Bias(%), CR$^{(d)}$, and VR denote the median percent bias, 95% confidence interval coverage rate of multivariate delta method, and mediation variance ratio, respectively. The coverage rates outside the 95% confidence boundary, i.e., $q \pm 1.96 \times \sqrt{\frac{q(1-q)}{B}}$, were highlighted in bold, where q denotes the nominal confidence interval threshold (95%) and B denotes number of replication (5,000). The median percent bias was calculated as the median of the ratio of bias to the true value over 5,000 replications, i.e., Bias(%) $= median(\frac{\hat{p}-p}{p}) \times 100\%$, where p denotes the true value of the causal mediation measure, and $\hat{p}$ is the point estimate of the simulated causal mediation measure. The median variance ratio is defined by the ratio of median delta-method variance estimators across 5,000 replications to the empirical variance of causal mediation measure estimates from the 5,000 replications.

Table 8: Basic characteristics of MaxART (Khan et al., 2020) participants at time of study enrollment

	Standard of Care (n=1014)	EAAA (n=717)	Overall (n=1731)
**Age group (years) n(%)**			
$[18, 20)$	21 (2.0%)	14 (1.9%)	35 (2.0%)
$[20, 30)$	347 (34.2%)	251 (35.0%)	598 (34.5%)
$[30, 40)$	325 (32.0%)	276 (38.4%)	601 (34.7%)
$[40, 50)$	187 (18.4%)	114 (15.9%)	301 (17.3%)
$[50, 60)$	92 (9.0%)	46 (6.4%)	138 (7.9%)
≥ 60	42 (4.1%)	16 (2.2%)	58 (3.3%)
**Sex n(%)**			
Male	303 (29.8%)	306 (42.6%)	609 (35.1%)
**level of clinic n (%)**			
Hospital	204 (20.1%)	176 (24.5%)	380 (21.9%)
Clinic with maternity	148 (14.6%)	98 (13.6%)	246 (14.2%)
Clinic without maternity	662 (65.2%)	443 (61.7%)	1105 (63.8%)
**Treatment support n (%)**			
Yes	988 (97.4%)	697 (97.2%)	1685 (97.3%)
**Marital status n(%)**			
Married	517 (50.9%)	349 (48.6%)	866 (50.0%)
Divorced/Widowed	69 (6.8%)	35 (4.8%)	104 (6.0%)
Single	413 (40.7%)	315 (43.9%)	728 (42.0%)
**Eduation n(%)**			
Illiterate/Primary	286 (28.2%)	222 (30.9%)	508 (29.3%)
Secondary	216 (21.3%)	188 (26.2%)	404 (23.3%)
High School	209 (20.6%)	107 (14.9%)	316 (18.2%)
Tertiary	18 (1.7%)	17 (2.3%)	35 (2.0%)
**BMI (kg/m2) n(%)**			
< 18.5	38 (3.7%)	32 (4.4%)	70 (4.0%)
$[18.5, 25)$	474 (46.7%)	383 (53.4%)	857 (49.5%)
$[25, 30)$	255 (25.1%)	154 (21.4%)	409 (23.6%)
≥ 30	214 (21.1%)	125 (17.4%)	339 (19.5%)
**CD4 (cells/ul) n(%)**			
< 350	353 (34.8%)	355 (49.5%)	708 (40.9%)
$[350, 500]$	221 (21.7%)	145 (20.2%)	366 (21.1%)
> 500	341 (33.6%)	159 (22.1%)	500 (28.8%)
**WHO stage n(%)**			
I	526 (51.8%)	407 (56.7%)	933 (53.9%)
II	143 (14.1%)	165 (23.0%)	308 (17.7%)

Table 8: Basic characteristics of MaxART (Khan et al., 2020) participants at time of study enrollment

	Standard of Care (n=1014)	EAAA (n=717)	Overall (n=1731)
III or IV	118 (11.6%)	96 (13.3%)	214 (12.3%)
**Viral load (copies/ml) n (%)**			
5000	235 (23.1%)	124 (17.2%)	359 (20.7%)
$[5000, 30000]$	214 (21.1%)	139 (19.3%)	353 (20.3%)
> 30000	501 (49.4%)	367 (51.1%)	868 (50.1%)
**clinic volume n (%)**			
Low: <median	549 (54.1%)	107 (14.9%)	656 (37.9%)
High: ≥median	465 (45.8%)	610 (85.0%)	1075 (62.1%)
**Time from HIV test positive to enrollment n (%)**			
< 1 yr	516 (50.8%)	497 (69.3%)	1013 (58.5%)
$[1, 3]$ yrs	259 (25.5%)	106 (14.7%)	365 (21.0%)
> 3 yrs	236 (23.2%)	109 (15.2%)	345 (19.9%)
**Screened for TB symptoms n(%)**			
Yes	91 (8.9%)	45 (6.2%)	136 (7.8%)

3 Web Figures

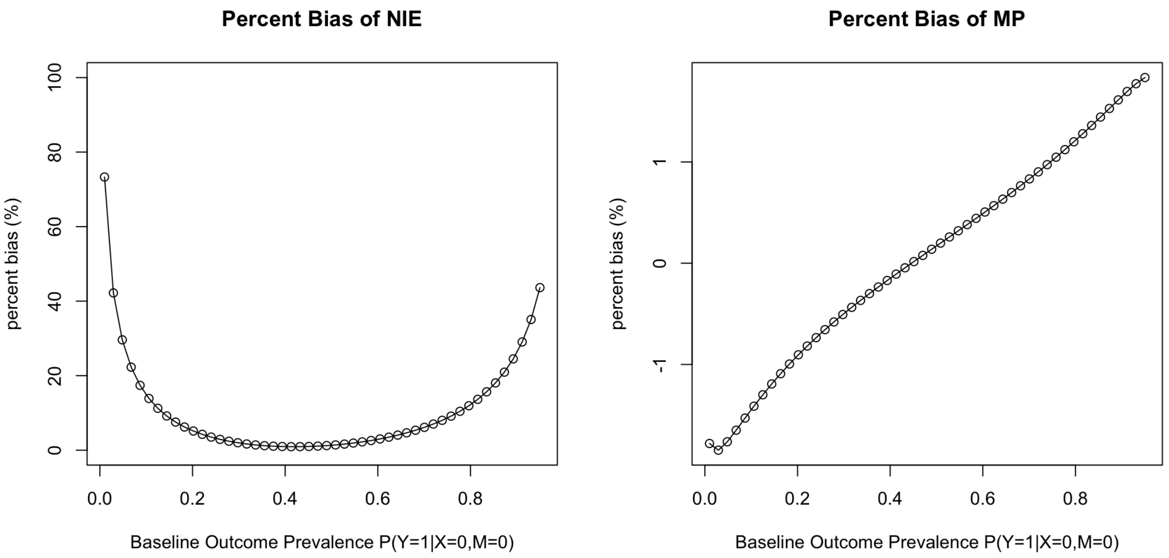

Figure 1: Percent bias for the probit approximation method in calculating the NIE and MP in Case #3, where TE=log(1.5), MP=0.5 and baseline outcome prevalence ranged from 0.01 to 0.95.

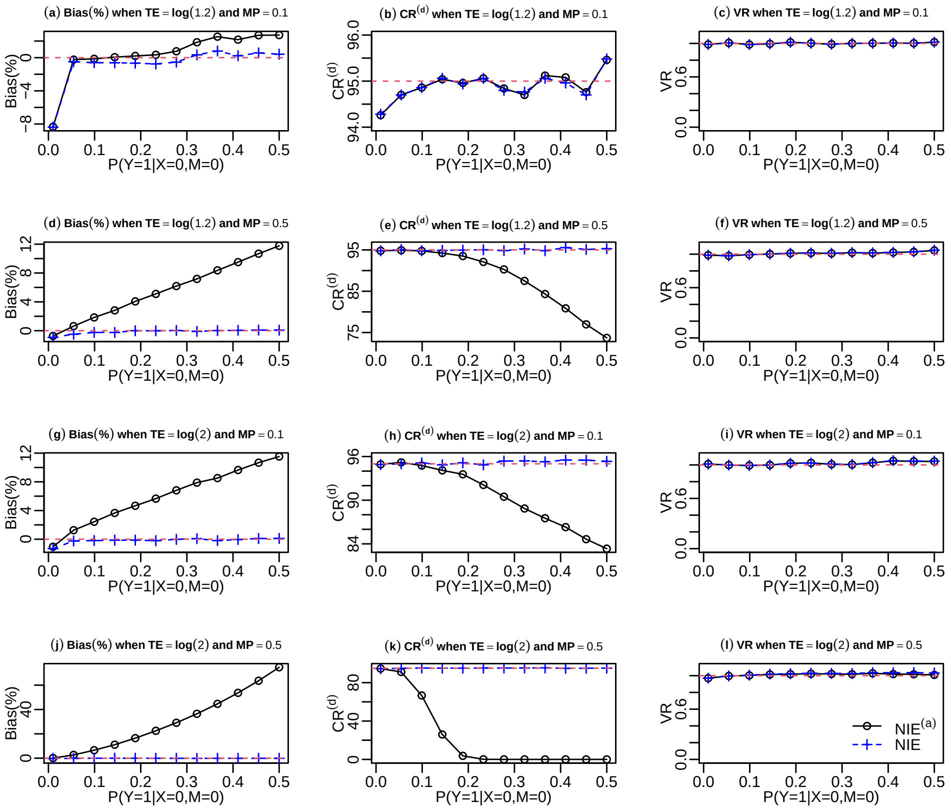

Figure 2: Performance of NIE$^{(a)}$ (black line) estimates and NIE estimates (blue dotted line) when changing baseline outcome prevalence from 1% to 50% in Case #4, where sample size is 20,000. Bias(%), CR$^{(d)}$, and VR denote the percent bias, coverage rate by the multivariate delta method, and variance ratio. Upper row: results for TE=log(1.2) and MP=0.1; second row: results for TE=log(1.2) and MP=0.5; third row: results for TE=log(2) and MP=0.1; bottom row: results for TE=log(2) and MP=0.5.

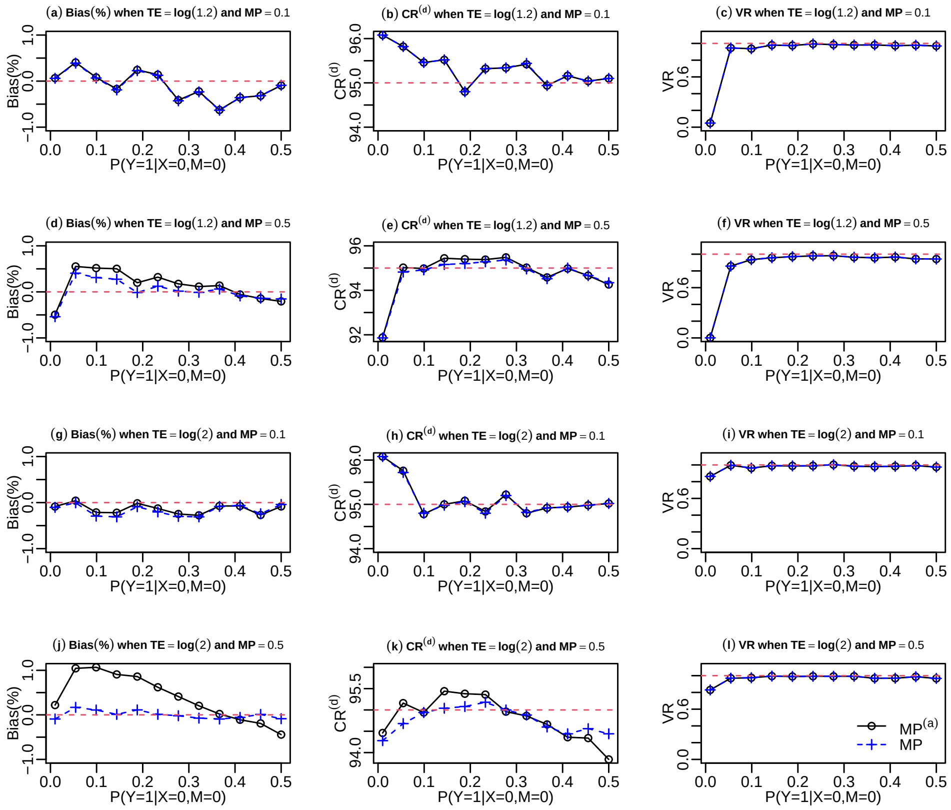

Figure 3: Performance of MP$^{(a)}$ (black line) estimates and MP estimates (blue dotted line) when changing baseline outcome prevalence from 1% to 50% in Case #3, where sample size is 20,000. Bias(%), CR$^{(d)}$, and VR denote the percent bias, coverage rate by the multivariate delta method, and variance ratio. Upper row: results for TE=log(1.2) and MP=0.1; second row: results for TE=log(1.2) and MP=0.5; third row: results for TE=log(2) and MP=0.1; bottom row: results for TE=log(2) and MP=0.5.

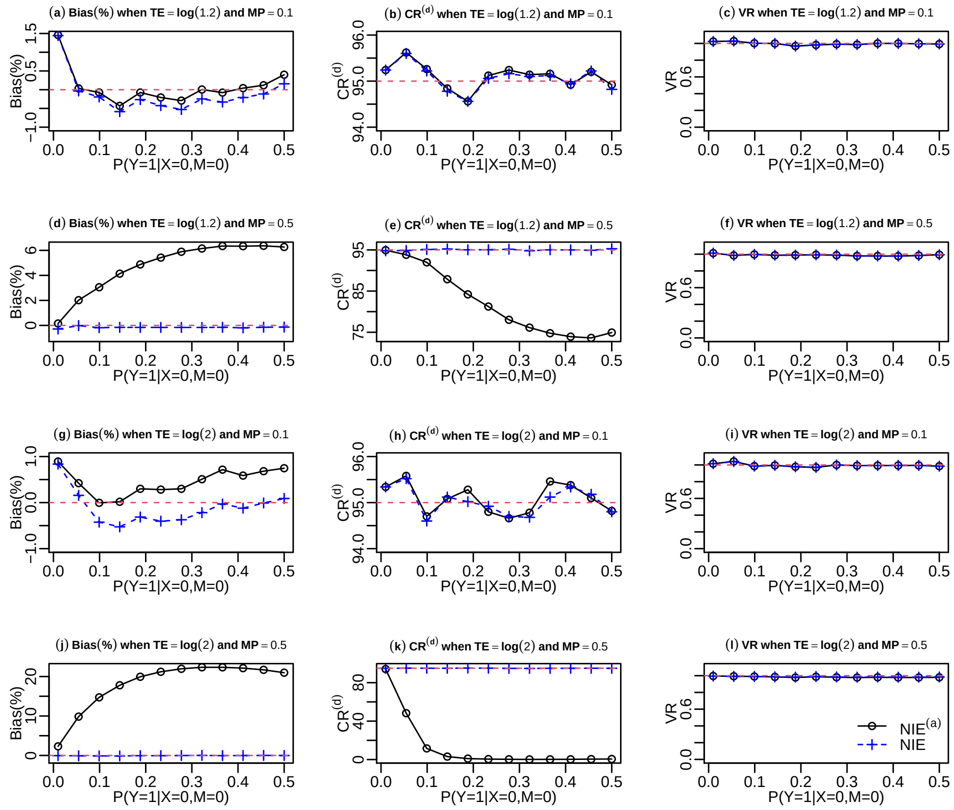

Figure 4: Performance of NIE$^{(a)}$ (black line) estimates and NIE estimates (blue dotted line) when changing baseline outcome prevalence from 1% to 50% in Case #3, where sample size is 20,000. Bias(%), CR$^{(d)}$, and VR denote the percent bias, coverage rate by the multivariate delta method, and variance ratio. Upper row: results for TE=log(1.2) and MP=0.1; second row: results for TE=log(1.2) and MP=0.5; third row: results for TE=log(2) and MP=0.1; bottom row: results for TE=log(2) and MP=0.5.
